# Genetic Ablation of a Female-Specific Apetala 2 Transcription Factor Blocks Oocyst Shedding in Cryptosporidium parvum

**DOI:** 10.1128/mbio.03261-22

**Published:** 2023-02-14

**Authors:** Jayesh Tandel, Katelyn A. Walzer, Jessica H. Byerly, Brittain Pinkston, Daniel P. Beiting, Boris Striepen

**Affiliations:** a Department of Pathobiology, School of Veterinary Medicine, University of Pennsylvania, Philadelphia, Pennsylvania, USA; b Center for Tropical and Emerging Global Diseases, University of Georgia, Athens, Georgia, USA; Albert Einstein College of Medicine

**Keywords:** AP2, *Cryptosporidium*, apicomplexan parasites, intestine, sex, transcription

## Abstract

The apicomplexan parasite *Cryptosporidium* is a leading global cause of diarrheal disease, and the infection poses a particularly grave threat to young children and those with weakened immune function. Infection occurs by ingestion of meiotic spores called oocysts, and transmission relies on fecal shedding of new oocysts. The entire life cycle thus occurs in a single host and features asexual as well as sexual forms of replication. Here, we identify and locus tag two Apetala 2-type (AP2) transcription factors and demonstrate that they are exclusively expressed in male and female gametes, respectively. To enable functional studies of essential genes in Cryptosporidium parvum, we develop and validate a small-molecule-inducible gene excision system, which we apply to the female factor AP2-F to achieve conditional gene knockout. Analyzing this mutant, we find the factor to be dispensable for asexual growth and early female fate determination *in vitro* but to be required for oocyst shedding in infected animals *in vivo*. Transcriptional analyses conducted in the presence or absence of AP2-F revealed that the factor controls the transcription of genes encoding crystalloid body proteins, which are exclusively expressed in female gametes. In C. parvum, the organelle is restricted to sporozoites, and its loss in other apicomplexan parasites leads to blocked transmission. Overall, our development of conditional gene ablation in C. parvum provides a robust method for genetic analysis in this parasite that enabled us to identify AP2-F as an essential regulator of transcription required for oocyst shedding and transmission.

## INTRODUCTION

Parasites rely on complex life cycles to colonize a variety of hosts, organs, and tissues, to massively amplify their numbers in the course of infection, and to enable transmission between hosts. The life cycles are defined by developmental progression through a series of distinct morphological stages, each highly adapted to specific environments and tasks. Examples include the formation of chronic tissue cysts for spread via carnivory and aggregation in the salivary glands of blood-feeding insects to enable vector-borne transmission. For members of the protist phylum Apicomplexa, these life cycles also alternate phases of clonal amplification through asexual cell division and sexual processes characterized by differentiated male and female gametes, fertilization, genomic recombination, and meiosis (1). Often, life cycle completion requires transmission between multiple hosts. In the case of Plasmodium falciparum, the causative agent of the most dangerous form of malaria, infection of humans and *Anopheles* mosquitoes is required to complete the life cycle. In contrast, *Cryptosporidium* has a much simpler single-host life cycle, where both asexual and sexual stages are found in the same host and the same tissue (2).

*Cryptosporidium* is one of the most important causes of severe diarrheal disease in a variety of epidemiological settings around the world (3–5). Cryptosporidiosis is life-threatening in immunosuppressed individuals, such as those with HIV-AIDS and transplant recipients. Immunocompetent individuals are susceptible, but the infection is self-limiting. In the United States, *Cryptosporidium* is responsible for 50% of disease outbreaks linked to recreational water use and is, overall, the most important cause of waterborne illness (5–7). Effective treatment is lacking, and more potent drugs are urgently needed (3, 4). An important lesson for *Cryptosporidium* drug development from recent advances in the search for antimalarials is to broaden the attack over the entire life cycle (8). In *Cryptosporidium* this is of even greater importance, as sustained infection is believed to be fed by successive cycles of autoinfection (8, 9). Currently, there is no vaccine to prevent cryptosporidiosis, but there is evidence that children develop protective natural immunity (10). The sexual phase of the life cycle offers attractive targets for vaccination, and both vaccine and drug development depend on a better understanding of the complete life cycle.

*Cryptosporidium* oocysts ingested with water or food release motile sporozoites into the intestinal tract which proceed to invade enterocytes, where they occupy an intracellular but extracytoplasmic apical niche (11, 12). Asexual replication (merogony) results in release of a new generation of motile and invasive forms, leading to the rapid spread of the parasite through the intestine. An intrinsic program of developmental commitment then leads to differentiation into male (micro) and female (macro) gametes (13). Male gametes egress to fertilize female gametes, which remain in their host cell. The resulting intracellular zygote undergoes meiosis and develops into a new oocyst. Once released from its host cell, either the oocyst is shed with the feces, resulting in transmission, or sporozoites emerge to reinitiate the cycle within the same host. Oocysts with a distinct wall structure have been morphologically described (9), and this has been suggested to reflect adaptation to differential transmission fates.

In the human cell line HCT-8, C. parvum initially vigorously amplifies through three rounds of asexual replication. After 48 h, parasites differentiate into gametes in a synchronous and highly predictable fashion (2, 13–15), and they do so directly from 8N type 1 meronts. However, while gametes form and meet in HCT-8 culture, fertilization fails, no new oocysts are formed, and parasites cease to proliferate (2). Longer-term proliferation has been achieved in stem cell-derived culture models, coinciding with sex and oocyst formation (16–18). This may suggest that *Cryptosporidium* sex contributes to continued infection of a single host in addition to its well-established role in transmission between hosts.

Progression through the apicomplexan life cycle is accompanied by dramatic changes in gene expression that often are the consequence of regulation at the level of transcriptional initiation (19); however, there are also examples of control through translational repression (20, 21). Initiation is governed by promoter elements immediately 5′ of apicomplexan genes that serve as landing spots for the transcription machinery and its regulatory components, and sequence-specific interactions between promoters and different transcription factors enable stage-specific expression patterns. Apetala-2-type (AP2) DNA binding proteins (22), in particular, have emerged as transcriptional modulators with central roles in apicomplexan life cycle progression. They have been linked to developmental regulation of gametocytes (23, 24), liver stages (25), ookinetes (26), and sporozoites (27) in *Plasmodium* spp. and tissue cyst formation in Toxoplasma gondii (28). The C. parvum genome encodes 16 putative ApiAP2 proteins (29, 30), some of which appear transcribed in a stage-specific fashion (2).

Here, we report the identification and analysis of two AP2 proteins that are exclusively expressed in male or female gametes, respectively. We found cgd4_1110, which encodes the female-specific AP2-F, to be refractory to targeted disruption of the locus. To enable genetic studies of essential genes, we established small-molecule-inducible Cre recombinase-mediated gene ablation. Using this model, we engineered mutant parasites in which the expression of cgd4_1110 is conditional and can be controlled with a small molecule. We tested the impact of loss of the protein on parasite development and overall infection *in vitro* and *in vivo*.

## RESULTS

### The AP2 DNA binding protein cgd4_1110 is exclusively expressed in female gametes.

**FIG 1:**
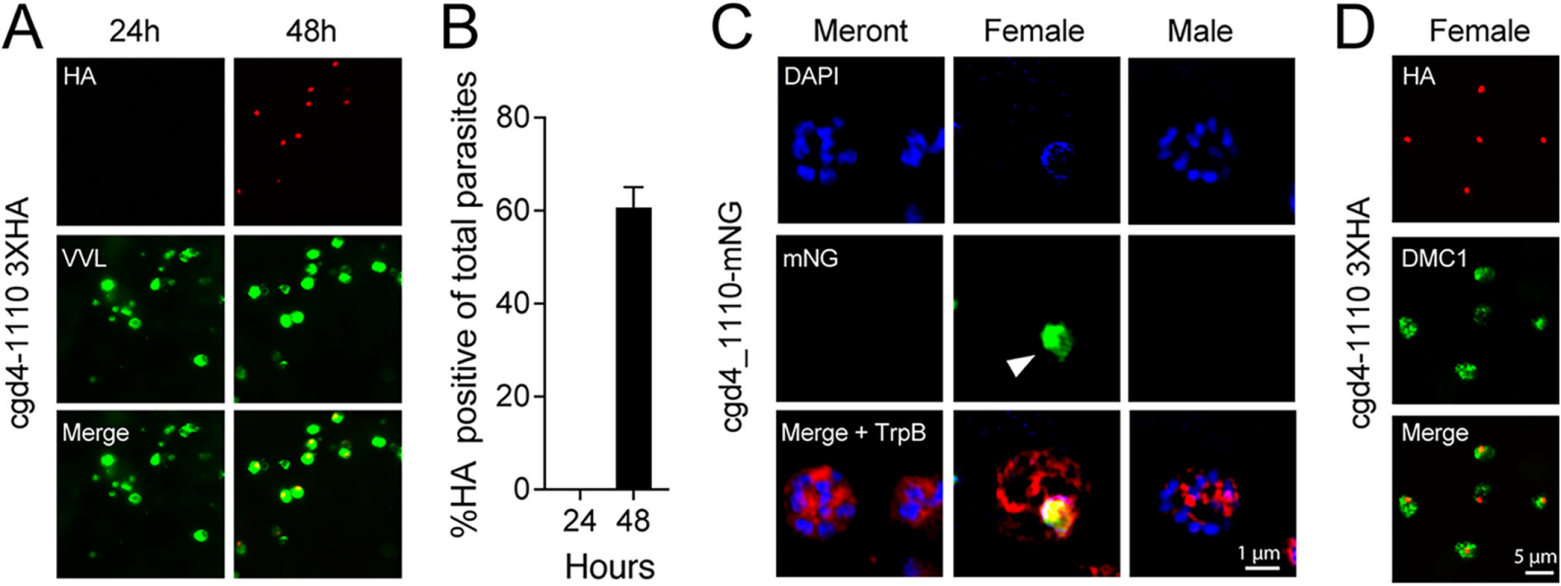
C. parvum cgd4-1110 encodes an AP2 DNA binding protein that is expressed exclusively in female gametes. (A) The locus of cgd4_1110 was modified to append a 3xHA epitope tag to the coding sequence. The resulting parasite strain was used to infect HCT-8 cell cultures and analyzed by immunofluorescence assays (IFA) at 24 and 48 h postinfection. The transgene was detected with antibodies to HA (red), and all parasites were labeled with *Vicia villosa* lectin (VVL, green). (B) Quantification of HA staining as a proportion of all parasites. (C) IFA of cultures infected with cgd4_1110-mNG showing asexual meronts, a female gamete, and a male gamont at 48 h. mNeon green (mNG) fluorescent protein is shown in green, DNA stained with DAPI in blue, and TrpB (labeling all parasites) in red. The single nucleus of the female gamete is highlighted with an arrowhead. (D) cgd4_1110-3xHA (red) expressing parasites stained for the female marker DMC1 (note that all HA-positive parasites are also DMC1 positive).

Little is known about the transcriptional control of life cycle progression in *Cryptosporidium* and the factors that orchestrate the commitment to, and the execution of, different developmental cell fates. However, the C. parvum genome encodes numerous AP2 DNA binding proteins, homologs of which have been linked to stage-specific gene expression control in the related parasite *Plasmodium* (19). Analysis and experimental manipulation of these factors thus may provide insight into the *Cryptosporidium* life cycle. Initial PCR screening at different times of culture suggested that some of these factors themselves may underlie transcriptional control across the life cycle (29, 30). We recently used engineered reporter parasites to isolate specific stages of *Cryptosporidium* and carried out comparative transcriptomics by transcriptome sequencing (RNA-seq) (2). This analysis suggested five AP2s to be preferentially transcribed in sexual stages (cgd1_3520, cgd2_3490, cgd4_1110, cgd6_2670, and cgd8_810). We initially focused on cgd4_1110, which appeared to be transcribed in female gametes, and tagged the gene to yield C-terminal translational fusion with either an mNeon-green reporter, or a triple hemagglutinin (HA) epitope tag introduced into the native locus by Cas9-mediated homologous recombination (31) (see Fig. S1 in the supplemental material for maps and PCR validation). cgd4_1110-3xHA parasites were used to infect HCT-8 cultures, which were subsequently fixed at 24 and 48 h postinfection, stained with an HA-specific antibody, and counterstained with Vicia villosa lectin, which recognizes glycan epitopes found on all C. parvum stages. No HA staining was observed at 24 h, a time point at which only asexual forms are found. However, at 48 h, when differentiation to gametes had occurred, 60.7% (±4,4%) of parasites were HA positive (Fig. 1A and B). Using cgd4_1110-mNG parasites, we similarly found label only at the 48-h time point. Relying on nuclear morphology to distinguish stages (2, 13) at this time point, we detected the reporter in female gametes, but not in asexual meronts or in male gamonts (Fig. 1C). Consistent with the presumptive function of a DNA-binding protein, the label coincided with the single nucleus of the female gamete (arrowhead, Fig. 1C). To further validate this finding using a molecular marker, we infected cells with cgd4_1110-HA parasites and stained them with antibodies to HA and the meiotic recombinase DMC1, which was recently shown to be restricted to female gametes (15). We observed HA staining exclusively in DMC1-positive cells (Fig. 1D).

10.1128/mbio.03261-22.1 FIG S1 Integration maps and validation PCR of all strains engineered. Diagnostic PCR products are shown as inverted arrows. Note that maps are not to scale and that the primers indicated here are listed in supplemental Data Set S2. Download FIG S1, TIF file, 3.8 MB. Copyright © 2023 Tandel et al. 2023 Tandel et al. https://creativecommons.org/licenses/by/4.0/ This content is distributed under the terms of the Creative Commons Attribution 4.0 International license.

### Male gamonts transiently express the AP2 protein cgd6_2670.

**FIG 2:**
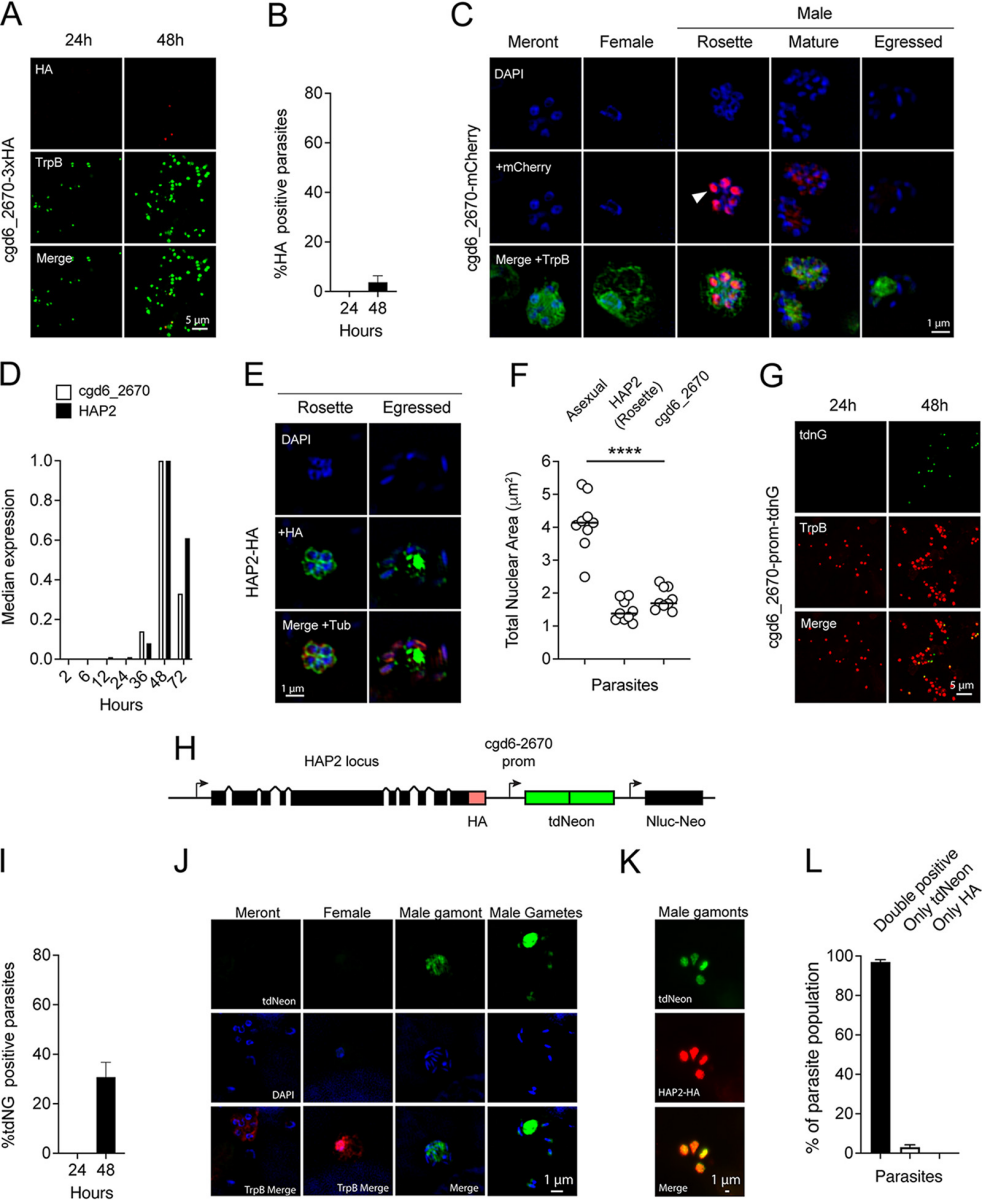
C. parvum cgd6_2670 encodes a cell cycle-restricted male gamont-specific AP2 protein. (A) A 3xHA tag was introduced into the cgd6_2670 locus, and the resulting parasites were subjected to IFA after growth in HCT-8 cultures for the indicated time. (B) Quantification of HA-positive parasites as a fraction of all parasites (TrpB). (C) Superresolution images showing parasites expressing a cgd6_2670-mCherry fusion protein (red) counterstained with TrpB (green). Note strong nuclear staining of parasite nuclear rosettes and weak labeling of mature male gamonts. (D) mRNA abundance for cgd6_2670 and HAP2 (qPCR data from reference 32). (E) IFA of HAP2-HA-expressing parasites showing labeled parasites with nuclear rosettes. (F) Quantification of the nuclear area (based on DAPI staining in IFAs) for asexual meronts and nuclear rosette stages stained for cgd_2670 or HAP2. (G) Expression of the tdNeon fluorescent reporter under the control of the cgd6_2670 promoter at the indicated times. (H) Schematic view of the HAP2 locus of the strain engineered for panels G to K. Note that this parasite simultaneously reports on the expression of HAP2 and cgd6_2670 but is released from posttranslational control of cgd6_2670. (I) Quantification of mNeon positivity over time. (J) IFA analysis of different stages. Note the green fluorescence in immature and mature male gametes. (K) IFA of four male gamonts (note different scale) labeled with tdNeon and HA antibody for HAP2; (L) quantification (*n* = 3, bars show the mean and standard deviation).

Previous transcriptional profiling work by us and others suggested that, due to the timing of expression, cgd6_2670 might also be a female-specific AP2 (2, 30). To further test this, the locus was tagged with a triple HA epitope or an mCherry reporter, and cultures infected with the resulting parasite strains were fixed at different time points. Upon staining, we observed fluorescence exclusively at the 48-h time point, as expected (Fig. 2A); however, compared to cgd4_1110, this staining was limited to a much smaller proportion of the overall parasite population (Fig. 2B). Analysis of the mCherry strain showed nuclear fluorescence exclusively in parasites with 4 or 8 round nuclei, which appeared clustered into a rosette at the center of intracellular parasite stages (Fig. 2C). No labeling was associated with identifiable asexual stages or with female gametes, but faint cytoplasmic staining was notable in mature male gamonts. The temporal pattern of cgd6_2670 transcription matches that of the male fusion protein HAP2 (Fig. 2D). We therefore studied parasites engineered to express an HA-tagged version of HAP2 (2, 32). HAP2 is found as a characteristic dot at the apex of mature male gametes and is perinuclear in immature gamonts, likely reflecting protein in the endoplasmic reticulum *en route* through the secretory pathway (Fig. 2E). Importantly, these immature stages showed centrally rosetted nuclei. We quantified the nuclear area of these stages (1.46 ± 0.32 μm^2^) and found it similar to that of cgd6_2670-positive rosette stages (1.829 ± 0.33 μm^2^) but significantly different from that of asexual meronts (Fig. 2F; 4.12 ± 0.83 μm^2^; *P* < 0.0001). The nuclear morphology of these stages is reminiscent of developing male gamonts recently observed by time lapse imaging (15). Next, we engineered parasites to express a tdNeon reporter under the control of the cgd6_2670 promoter to mark all stages in which this gene is transcribed. In contrast to our previous experiments, we did not fuse the reporter to the AP2 protein itself, thus releasing it from posttranslational control through development (see Fig. 2H). We introduced this construct into the HAP2 locus, adding an HA tag to specifically identify males. When studying the resulting transgenic strain, we again found fluorescence to be restricted to the 48-h time point, but now observable in higher numbers, matching those previously observed for male gamonts (Fig. 2G to J) (2). Importantly, essentially all parasites expressing tdNeon were also positive for the male marker HAP2-HA (Fig. 2K and L). We conclude that the two AP2 proteins characterized here are expressed in a female (cgd4_1110) and early-male-specific (cgd6_2670) fashion and thus named them AP2-F and AP2-M, respectively.

### Conditional gene ablation in Cryptosporidium parvum.

To understand the role of gamete-specific AP2 factors in *Cryptosporidium* life cycle progression, we next set out to disrupt their loci. An attempt to disrupt AP2-M was unsuccessful (data not shown). We next attempted to insert a drug marker into the N-terminal AP2-F DNA binding domain. This was unsuccessful in five attempts using three different guide RNAs. Parallel control experiments inserting an epitope tag without altering the coding sequence readily resulted in transgenic parasites (we note that we were able to disrupt the C-terminal DNA binding domain; this did not impact parasite growth or development, and we thus did not study this mutant any further; see Fig. S2). In C. parvum transgenesis, selection occurs in animals, and recovery of modified parasites requires the formation of oocysts, following the completion of the full life cycle (31). While failure to disrupt the locus is consistent with the essentiality of a gene, it is not proof, and we thus next sought to develop an experimental model to ablate genes in C. parvum conditionally. Multiple approaches have been adapted to apicomplexans for this purpose, including modulating transcription initiation (33), affecting the stability of the targeted mRNA or protein (34–37), or using recombinases to excise portions of the genome (38, 39).

10.1128/mbio.03261-22.2 FIG S2 The AP2-F locus is refractory to disruption of the N-terminal AP2 DNA binding site. (A) Map showing three different modifications of the AP2-F locus that were attempted here: deletion of the N-terminal or C-terminal AP2 DNA binding domain or C-terminal epitope tagging. (B) Luciferase measurements of feces collected from mice, infected with sporozoites transfected with the indicated vectors. Note that while viable parasites emerged from selection for AP2-F epitope tagging and KO of the C-terminal domain, that was not the case for the N-terminal deletion (which ablates essentially the entire gene). (C) PCR analysis demonstrating successful insertion into the portion of the AP2-F gene encoding the C-terminal AP2 DNA binding domain. Download FIG S2, TIF file, 1.4 MB. Copyright © 2023 Tandel et al. 2023 Tandel et al. https://creativecommons.org/licenses/by/4.0/ This content is distributed under the terms of the Creative Commons Attribution 4.0 International license.

**FIG 3:**
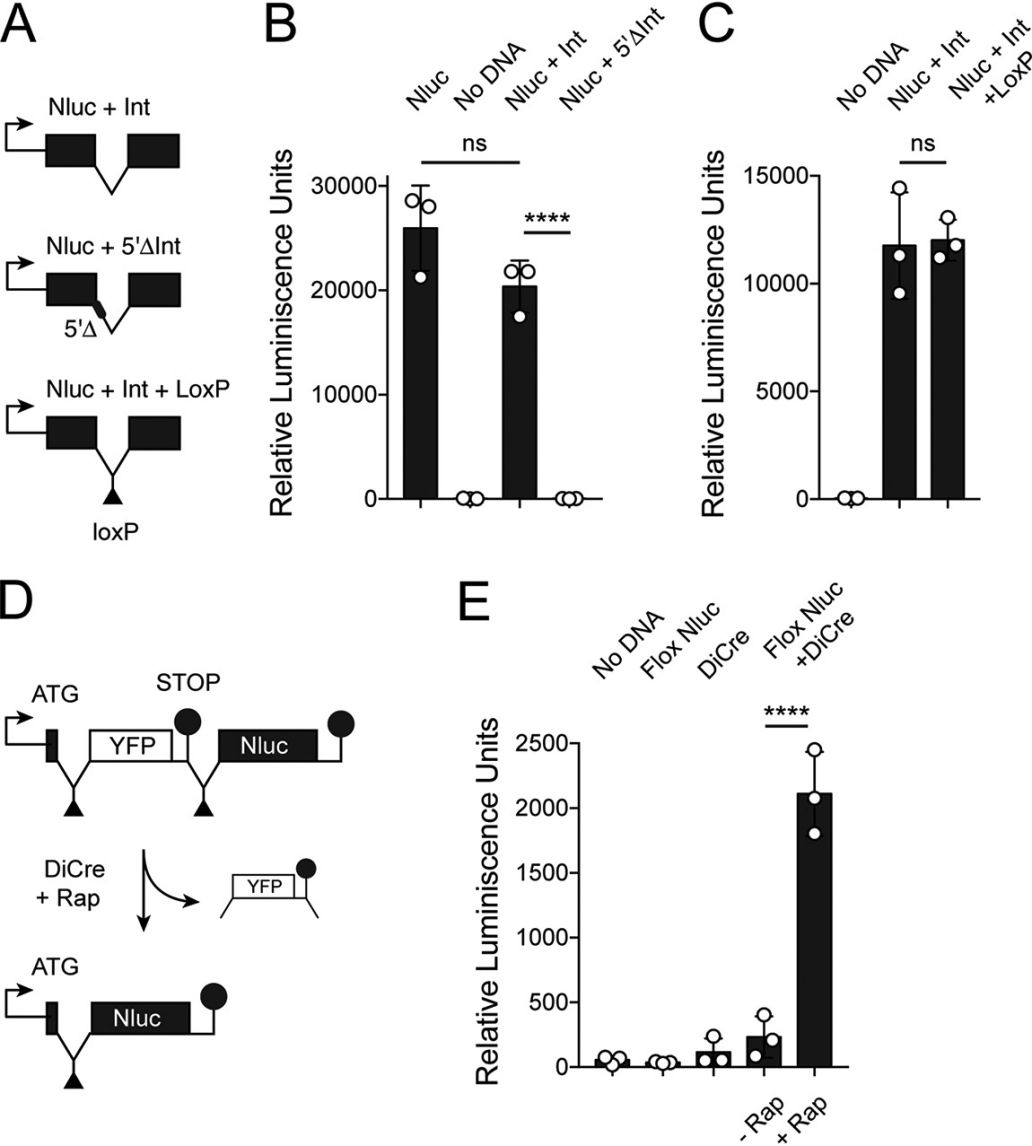
Cre-mediated DNA excision using a floxed intron sequence. (A) Schematic maps of Nluc reporters used in transient transfection experiments. (B and C) Sporozoites were transfected with the indicated constructs (see Materials and Methods for details) and used to infect HCT-8 cell cultures. Luciferase activity was measured after 48 h of culture. Note that introns and loxP sites within introns do not perturb Nluc expression. (D) Schematic outline of the transient Cre recombinase assay. Rapamycin (Rap) induction of Di-Cre results in excision of a floxed stop sequence (YFP) and expression of Nluc. (E) Transient transfection assays using the indicated constructs followed by luciferase measurement. Note induction of Nluc activity upon addition of rapamycin (+Rap) when DiCre and floxed reporter (Flox Nluc) plasmids were cotransfected. (B, C, and E) Each symbol represents one independent well, and the bar shows the mean and standard deviation. Significance was evaluated using an unpaired Student’s *t* test.

Here, we used a modified version of Cre-recombinase (DiCre) that, upon rapamycin induction, assembles an active enzyme from two fragments to excise gene segments flanked by loxP recognition sequences (38, 40). To enable the introduction of loxP sites into the coding sequence of genes without changing their protein products, we explored the use of introns. We searched the C. parvum genome for short introns and through preliminary experimentation settled on the 73-bp intron from cgd1_1320, which is expressed across the life cycle (32). To test the functionality of this intron in *trans*, we inserted it into the nanoluciferase (Nluc) coding region (Fig. 3A). Transient transfection of this reporter into sporozoites followed by infection of HCT-8 cells resulted in luciferase activity indistinguishable from that obtained with a continuous Nluc coding sequence (Fig. 3B). Faithful splicing was critical for this, as the activity was lost when the 5′ splice donor site of the intron was ablated (Fig. 3B; *P* < 0.0001). Next, loxP sequences were introduced at different positions of the intron, and luciferase activity was measured. We identified multiple positions that resulted in activity comparable to that of the native intron (Fig. 3C; see Fig. S3 for detail). We conclude that the intron is faithfully spliced when introduced in *trans*, and its splicing remains intact when loxP sites are incorporated.

10.1128/mbio.03261-22.3 FIG S3 The floxed artificial intron used in this study. The position of three loxP sites within the intron found in the C. parvum gene cgd1_1320 that tolerate insertion of a loxP site are shown. The loxP site shown in bold was used for subsequent constructs, and the insert below shows the loxP (uppercase) insertion in detail. Download FIG S3, TIF file, 0.5 MB. Copyright © 2023 Tandel et al. 2023 Tandel et al. https://creativecommons.org/licenses/by/4.0/ This content is distributed under the terms of the Creative Commons Attribution 4.0 International license.

Next, we constructed a plasmid that expresses two fragments of Cre recombinase under the control of the C. parvum enolase promoter, each fragment fused to FKBP12 or FRB, respectively, and separated by a viral translational skip sequence (40). To test whether this system results in rapamycin-inducible Cre activity in C. parvum, we designed a transient reporter assay. In our test construct, Nluc expression was disrupted by a floxed stop sequence containing a yellow fluorescent protein (YFP) reporter (Fig. 3D). Cre-mediated excision should remove the stop sequence and result in Nluc expression. Transfection of this floxed reporter, or DiCre alone, did not result in luciferase activity above a background control. However, when parasites were simultaneously transfected with both constructs and grown in culture medium containing 100 nM rapamycin, robust activity was detected, and this activity depended on the presence of rapamycin (Fig. 3E; *P* < 0.0001). Please note that rapamycin does not impede *Cryptosporidium* growth at the concentration used here. We tested a range of concentrations and determined the 50% inhibitory concentration (IC_50_) to be 8.3 μm (see Fig. S4).

10.1128/mbio.03261-22.4 FIG S4 Impact of rapamycin for *Cryptosporidium* growth in culture. HCT-8 cell cultures were infected with C. parvum expressing Nluc luciferase and grown in the presence of different concentrations of rapamycin for 48 h. Growth was measured by luciferase assay (each symbol shows the mean of three biological replicates, and the error bar shows the standard deviation; values were normalized with the highest measurement set to 100%). The IC_50_ was determined using Prism v.9 software and a nonlinear fit model shown as a line here. Concentrations above the IC_50_ are highlighted in red. Concentrations used in this study (100 nM) and lower are highlighted in blue, and we note that in the range used, rapamycin does not impact parasite growth. Download FIG S4, TIF file, 0.4 MB. Copyright © 2023 Tandel et al. 2023 Tandel et al. https://creativecommons.org/licenses/by/4.0/ This content is distributed under the terms of the Creative Commons Attribution 4.0 International license.

### Rapamycin-inducible gene excision from the *Cryptosporidium* genome.

**FIG 4:**
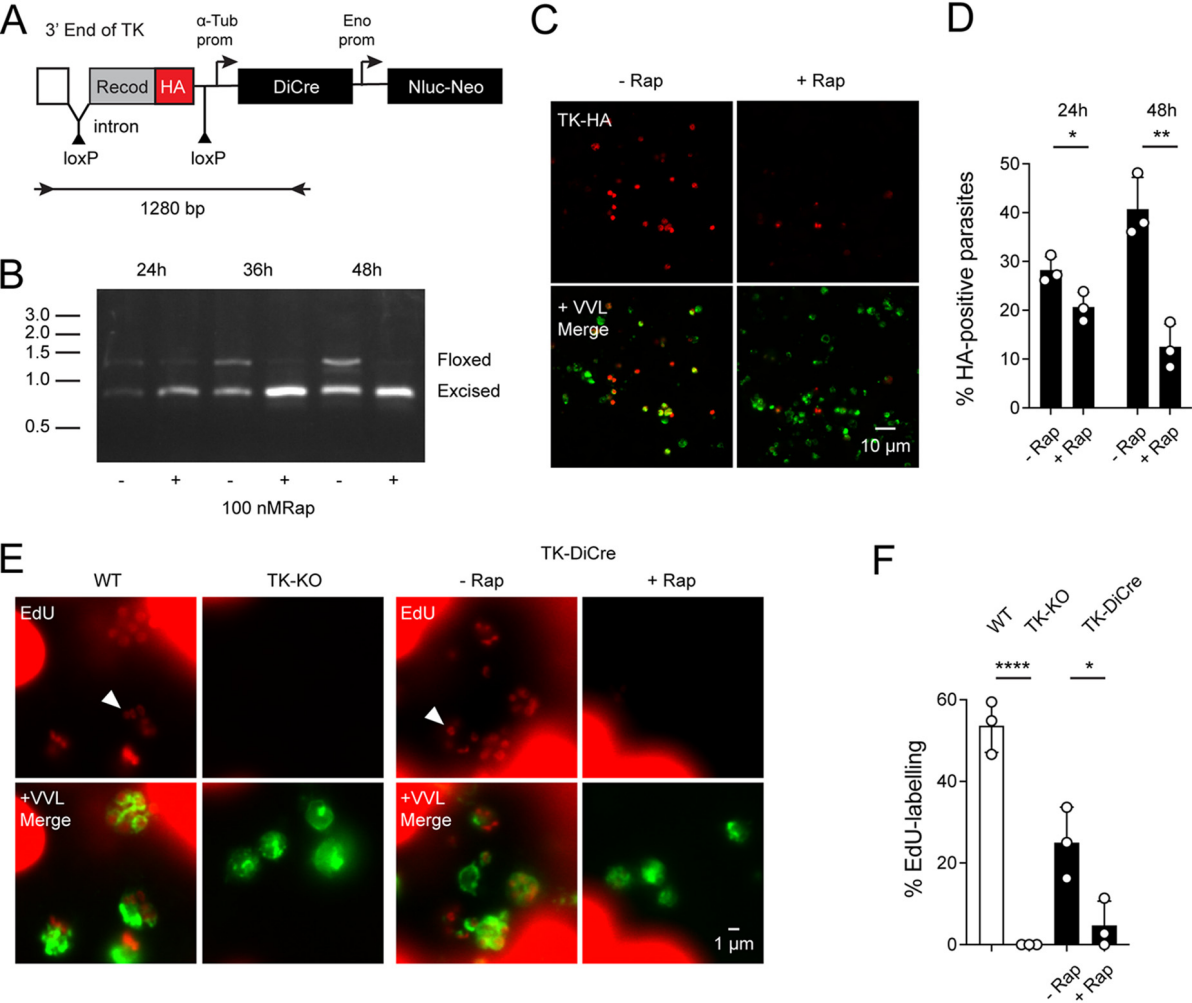
Inducible excision of a gene from the C. parvum genome. (A) Schematic view of the targeting construct used to modify the thymidine kinase (TK) locus. Note that the HA tag is located between loxP sites and will be excised due to Cre activity. (B) Modified parasites were used to infect HCT-8 cell cultures for the indicated times in the presence or absence of rapamycin. Genomic DNA was extracted and used for PCR analysis, and the resulting amplicons were detected and sized by gel electrophoresis; size markers are shown in 1,000 bp (kb). Diagnostic amplicons for the floxed and excised locus are highlighted. (C) Infected cultures grown in the presence or absence of rapamycin for 48 h prior to IFA and staining with antibody to HA (red) and VVL (green). (D) Quantification of IFA experiments showing HA-positive cells as a fraction of all parasites (labeled with VVL). (E) 5-Ethynyl-2′-deoxyuridine (EdU, red) incorporation assay, which requires TK activity; parasite nuclei are highlighted by arrowheads. Parasites were counterstained with VVL (note that the much larger host cells robustly incorporate EdU as well; this remains unchanged by rapamycin treatment). (F) Quantification of EdU incorporation comparing the wild type (WT), a mutant in which the entire locus was replaced with a marker gene (TK-KO), and the inducible mutant (TK-DiCre) grown in the presence or absence of rapamycin. Each symbol represents an independent culture; the bar shows the mean with standard deviation. Significance was evaluated using unpaired Student’s *t* test. (C and E) for additional images see Fig. S5A and B, respectively.

Next, we tested the ability of the system to excise floxed sequences from the parasite genome. We targeted the well-characterized nonessential thymidine kinase (TK) locus (41, 42), replacing the last 222 bp of the native sequence with a recodonized version preceded by an artificial intron carrying a loxP site (Fig. 4A). We also appended a 3XHA epitope tag to be able to detect the protein along with cassettes for expression of DiCre and the Nluc-Neo selection marker. PCR mapping confirmed successful modification of the TK gene (Fig. S1), and Sanger sequencing of the amplified insert demonstrated that the targeted region was flanked by loxP sites. Using primers flanking the floxed region, we detected two amplicons representing the floxed locus, prior to and following Cre-mediated excision (note that all primers used in this study are listed in supplementary Data Set S2). The initially faint smaller 743-bp band became more noticeable upon continuous passage of the strain, suggesting that stable expression of DiCre recombinase in this strain resulted in some leaky activity in the absence of rapamycin.

**FIG 5:**
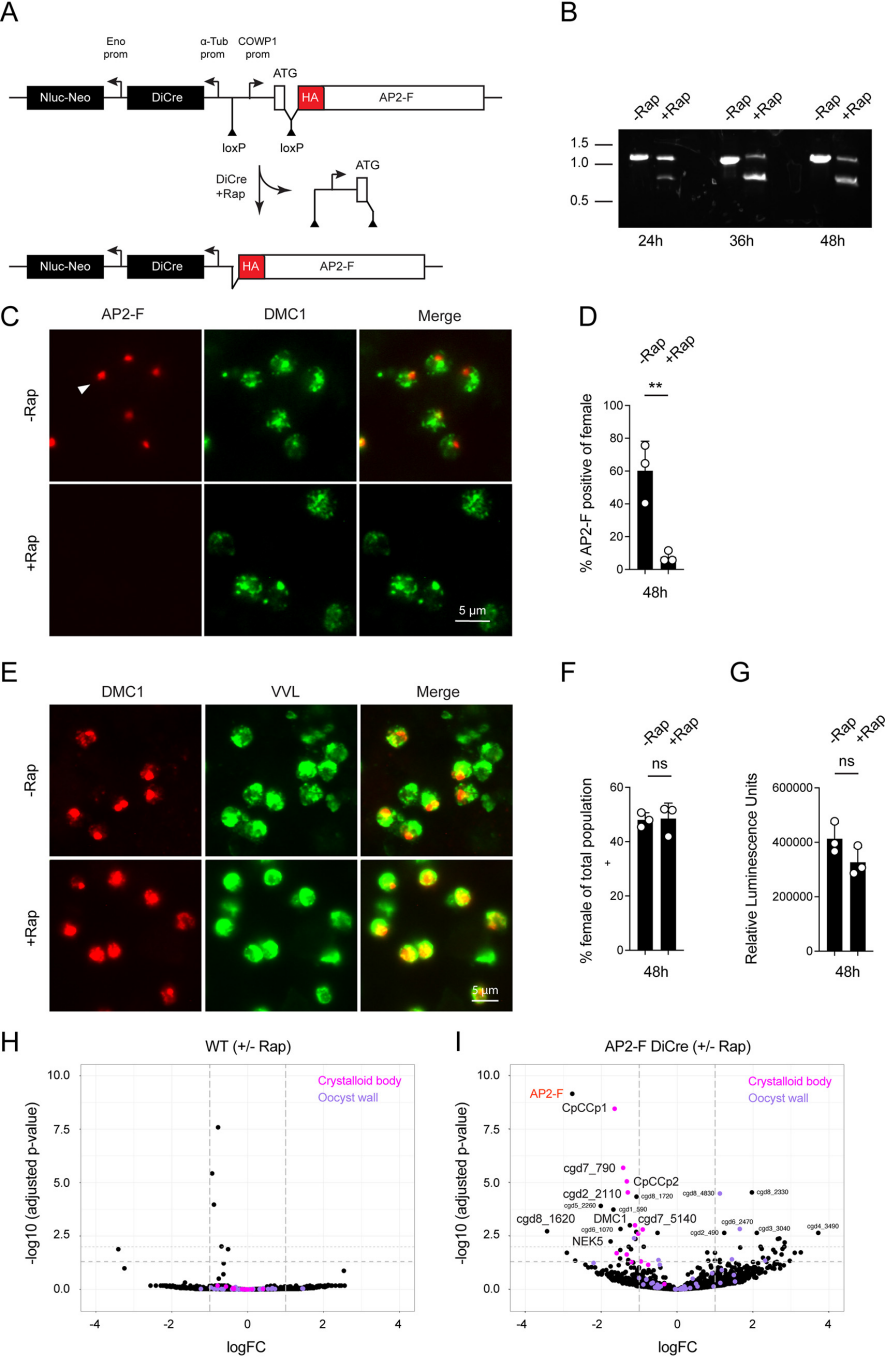
Conditional ablation of AP2-F expression by promoter excision. (A) Schematic view of the modified AP2-F locus prior to and after Cre-mediated excision. Note that the 3′ loxP site was introduced into the coding sequence within an artificial intron and that excision removes both the promoter and initiation codon. (B) Modified parasites were used to infect HCT-8 cell cultures for the indicated times in the presence or absence of rapamycin. Genomic DNA was extracted and used for PCR analysis, and the resulting amplicons were detected and sized by gel electrophoresis; size markers are shown in 1,000 bp (kb). Diagnostic amplicons are highlighted. (C) IFA after 48 h with or with rapamycin. AP2-F is labeled by HA (red, note staining of single nuclei), and female parasites were labeled with an antibody to DMC1 (green). (D) Quantification of AP2-F-positive parasites as a fraction of all female parasites at 48 h. (E and F) IFA scoring of the proportion of all AF2-F/DiCre parasites (VVL, green) that develop into female gametes (DMC1, red) after 48 h when grown with or without rapamycin. (G) Luciferase assay measuring growth of AP2-F/DiCre parasites after 48 h with or without rapamycin. Each symbol represents an independent culture, and the bar shows the mean with standard deviation. Significance was evaluated using an unpaired Student’s *t* test. (H and I) WT (H) or AP2-F/DiCre (I) parasites were used to infect HCT-8 cultures. After 48 h of growth, in the presence or absence of rapamycin, RNA was extracted and sequenced. Volcano plots show the relative abundance of transcript with and without treatment. Note the lack of change for the WT and enrichment of crystalloid body components versus oocyst wall components (highlighted in pink and purple, respectively). (C and E) For additional images see Fig. S5C and D, respectively.

10.1128/mbio.03261-22.5 FIG S5 Additional representative micrographs demonstrating the level and consequence of rapamycin-induced DiCre activity. (A) Inducible TK knockout (shown in Fig. 4C). Infected cultures grown in the presence or absence of rapamycin for 48 h prior to IFA and staining with antibody to HA (red) and VVL (green). (B) Inducible TK knockout (shown in Fig. 4E). 5-Ethynyl-2′-deoxyuridine (EdU, red) incorporation assay, which requires TK activity; parasite nuclei are highlighted by arrowheads. Parasites were counterstained with VVL. (C) AP2-F conditional knockout (shown in Fig. 5C). IFA after 48 h with or without rapamycin. AP2-F is labeled by HA (red; note staining of single nuclei), and female parasites were labeled with an antibody to DMC1 (green). (D). IFA scoring the proportion of all AP2-F/DiCre parasites (VVL, green) that develop into female gametes (DMC1, red) after 48 h when grown with or without rapamycin (Shown in Fig. 5E). Download FIG S5, TIF file, 5.1 MB. Copyright © 2023 Tandel et al. 2023 Tandel et al. https://creativecommons.org/licenses/by/4.0/ This content is distributed under the terms of the Creative Commons Attribution 4.0 International license.

We next infected HCT-8 cells with TK DiCre parasites, and cultures were harvested after 24, 36, and 48 h with or without rapamycin, and genomic DNA was isolated and analyzed by PCR for the presence of the floxed region (Fig. 4B). Rapamycin treatment resulted in progressive loss of the full-length band beginning at 24 h postinfection (hpi). We next scored parasites in immunofluorescence experiments using anti-HA antibody to detect the (floxed) epitope and *Vicia villosa* lectin (VVL) as counterstain for all parasites. As expected from the PCR mapping, we detected both tagged and untagged parasites in cultures grown in the absence of rapamycin. When rapamycin was added to cultures, we detected significant loss of HA staining (Fig. 4C and D; control versus rapamycin, 12.53% ± 4.67% after 48 h of rapamycin compared to 40.72% ± 6.5% in controls; *P* < 0.01). We also directly measured the biochemical activity of TK using 5-ethynyl-2′-deoxyuridine (EdU) incorporation. EdU incorporation relies on TK to activate the probe to the nucleotide level (31, 43). Wile-type (WT) parasites and a mutant lacking TK (31) were used as positive and negative controls, respectively. A total of 53.64% ± 6.5% of the WT parasites were EdU-positive, while no staining was observed in the TK knockout (KO) (Fig. 4E and F). In the TK DiCre strain 24.98% ± 8.7% of the untreated parasites were found to be EdU-positive, and this number dropped to 4.7% ± 5.94% upon rapamycin treatment (Fig. 4F, *P* < 0.05). We conclude that C. parvum genes tolerate a floxed intron retaining normal transcription and translation, and that treatment with rapamycin induces loss of the targeted gene and its encoded protein and activity. We also noted some leaky DiCre activity when targeting a nonessential gene.

### Inducible loss of AP2-F through promoter ablation.

We next sought to impose rapamycin-inducible ablation on the likely essential AP2-F gene. Our initial attempts to flox the entire gene while concurrently introducing cassettes for DiCre and selection marker failed. In our experience, targeting cassettes exceeding 5 kb in size often fail to recombine efficiently. We thus decided to flox the promoter and initiation codon, a much shorter, but nonetheless essential sequence (see schematic in Fig. 5A). To avoid undue recombination, we typically recodonize homologous sequences that are localized internally within the targeting cassette. This is not feasible for a promoter, and we therefore replaced the native AP2F promoter with a floxed promoter with a matching transcriptional profile. We chose the promoter of the *Cryptosporidium* oocyst wall protein 1 (COWP1), which is active exclusively in female gametes (2), and we also added an N-terminal 3XHA epitope tag. Transfection with this construct produced viable drug-resistant parasites, and PCR mapping and Sanger sequencing confirmed the modification, indicating that the promoter swap was tolerated (Fig. S1). HCT-8 cells were infected with the AP2-F floxed strain and incubated for 24, 36, and 48 h in the presence or absence of 100 nM rapamycin. PCR analysis of genomic DNA isolated from these cultures, using primers spanning both loxP sites, found a single band in parasites grown in the absence of rapamycin (Fig. 5B). Growth in the presence of rapamycin resulted in the appearance of a second smaller band, indicating promoter excision. To assess the impact of rapamycin treatment on the level of AP2-F protein, infected HCT-8 cultures were stained with antibodies to HA (AP2-F) and DNA meiotic recombinase 1 (DMC1, a marker for female gametes [15]; Fig. 5C). Rapamycin treatment resulted in significant loss of AP2-F staining, 7.62 ± 3.38% compared to 60.22% ± 18% in untreated controls (Fig. 5D, *P* < 0.01), but did not impact parasite growth in culture (Fig. 5G). As shown in Fig. 5E and F, loss of AP2-F did not result in loss of female parasites, suggesting that AP2-F does not act as a female commitment factor.

To test whether AP2-F affects gene expression and to identify genes modulated by the factor, we next performed mRNA sequencing experiments. Three biological replicates of WT and AP2-F-DiCre parasites were grown in HCT-8 cells with or without rapamycin for 48 h, RNA was extracted, and cDNA libraries were prepared for Illumina sequencing. Comparison of WT parasites treated with and without rapamycin yielded little difference in parasite gene expression (Fig. 5H), indicating that rapamycin alone did not induce a significant transcriptional response. Upon addition of rapamycin to the AP2-F-DiCre strain, we noted a reduction of transcripts for AP2-F itself by greater than 2.5-fold (Fig. 5I, highlighted in red). A total of 15 additional genes showed significantly decreased expression, and 17 genes increased in RNA abundance (see Fig. 5I and supplemental Data Set S1). Of the 16 genes with reduced expression upon treatment, 14 showed tight specificity for female gametes in a previous study (2), while only 6 of 17 with increased expression were restricted to female gametes. Most prominent among the female-specific transcripts was a cluster of crystalloid body proteins (all proteins linked to this sporozoite-specific organelle in a recent proteomic study [A. Guérin and B. Striepen, unpublished observation] are highlighted in magenta in Fig. 5H and I). Two proteins involved in meiosis, DMC1 and NIMA kinase 5, also showed a reduction, as did a DNA polymerase. We note that other abundant classes of female proteins, such as those that make up the oocyst wall proteome (highlighted in purple), remained unchanged, suggesting specificity for a subset of female genes.

### Reduction of AP2-F results in the reduction of oocyst shedding *in vivo*.

**FIG 6:**
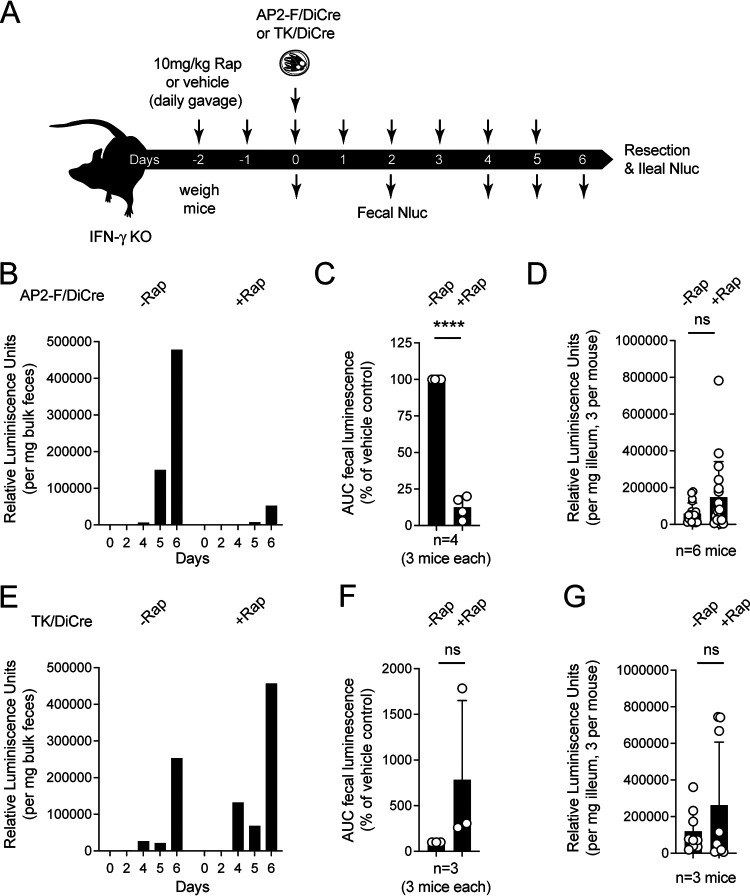
Ablation of AP2-F in infected animals leads to reduced oocyst shedding. (A to G) Schematic outline (A) of treatment and sampling for infection of IFN^–/–^ mice with AP2-F/DiCre (B to D) or TK/DiCre (E to G) parasites. (B and E) Luciferase measurements of feces collected from cages of three mice over the course of 6 days of infection. (C and F). Area under the curve for luciferase measurements as shown in panels B and E across 4 (C) and 2 (F) repeats. (D and G) Mice were euthanized on day 6 of infection, intestines were resected and flushed with buffer, and three ileal punch biopsies were taken per mouse and used in luciferase assays as a proxy for the intensity of intestinal infection. Symbols show individual mice or biopsies, and bars indicate the mean and standard deviation. Significance was determined by unpaired Student’s *t* test. Note that values in panel F that exceed 100% reflect immunosuppression under rapamycin leading to enhanced parasite growth.

Our initial KO experiments suggested AP2-F to be an essential gene, but while we noted changes in female transcription in culture upon rapamycin treatment, these changes did not result in obvious changes in either growth or differentiation over the development observable in HCT-8 cell culture (fertilization, meiosis, and oocyst formation and release do not occur in this system [2]). We thus considered that the processes governed by AP2-F, including the requirement for the crystalloid body, may manifest later in female development, and at a time point not well represented by *in vitro* culture. To test this, we conducted *in vivo* infection experiments in mice. Groups of three gamma interferon KO (IFN-γKO) mice were treated daily by gavage with 100 μL vehicle or 10 mg/kg rapamycin, beginning 2 days prior to infection with 10,000 AP2-F-DiCre strain oocysts (Fig. 6A). Nluc activity was monitored in mouse feces, and we observed significant reduction of oocyst shedding in mice treated with rapamycin compared to the vehicle control (Fig. 6B). We found this to be true in four independent biological experiments using groups of three mice each. Fig. 6C shows the area under the curve normalized for each matched group of treated and untreated mouse samples for all experiments (*P* < 0.0001). Mice were euthanized on day 6 in two of these experiments, intestines were resected, and Nluc activity was measured for three ileal punch biopsies per mouse. In contrast to fecal shedding, intestinal Nluc readings were not reduced but were moderately higher in treated mice (Fig. 6D). To measure the effects of rapamycin treatment alone, we used the TK-DiCre strain as a control (Fig. 6E to G; note that the TK gene is dispensable). We found rapamycin to be of no detriment to infection with these parasites, and luciferase readings were consistently higher upon treatment, regardless of whether we measured fecal or intestinal luciferase. This is likely due to the immunosuppressive effects of this drug (44). We conclude that AP2-F is critical to the formation and/or shedding of oocysts from infected animals but that it is not required for the growth of parasites in the intestinal tissue.

## DISCUSSION

New experimental tools have enabled a reevaluation of the *Cryptosporidium* life cycle. The picture emerging from these studies is that of a remarkably succinct developmental program, which relies on a single host and only three morphological stages and unfolds over the course of only 3 days (2, 13, 15, 16). However, the molecular mechanisms by which this program is controlled remain poorly understood. In this study, we identified and characterized two AP2 transcription factors that are exclusively expressed in gametes. We demonstrated AP2-M to be a male factor and, not as initially assumed, a female-specific protein. Interestingly, labeling for the protein is narrowly restricted to the mitotic phase of male development and lost prior to full maturation of the 16 male gametes. This is consistent with a model of the apicomplexan cell and developmental cycle driven by waves of gene expression, controlled by a succession of specific transcription factors (1, 24). Each phase is initiated by transcription and translation of the regulatory factor and terminated, at a defined point in the cell cycle, by degradation of that factor, thus yielding the field to the next transcription factor and the next wave of transcripts (45–47).

Genetic studies of developmental regulation in *Plasmodium* or *Toxoplasma* have been facilitated by the fact that the transition to sex or cyst formation, respectively, is not essential to the growth of the life cycle stages used to isolate mutants. In contrast, transgenesis in *Cryptosporidium* requires sporozoites, the products of sex (31). It is thus impractical to ablate transcription factors critical to life cycle progression. To overcome this limitation, we adapted a DiCre conditional gene excision system that can be triggered by rapamycin treatment (38, 40) and used it to disrupt the AP2-F gene. Typically, such systems are assembled in multiple steps that first introduce the recombinase and subsequently flank the target gene with loxP sites. The technical requirements of C. parvum transfection forced us to miniaturize the components into one cassette, to be delivered in a single genomic recombination event. Our strategy relied on short introns carrying loxP sites, and we showed that those are well tolerated and efficiently excised using two genes, TK, and AP2-F, as examples. To conditionally disrupt the large AP2-F gene, we replaced its promoter with a floxed promoter. Intergenic regions, including promoters, are very short in *Cryptosporidium* (48), offering attractive targets for excision. Critical to implementing this strategy is to identify a surrogate promoter that will preserve the timing and strength of expression of the native gene. Multiple transcriptomic data sets facilitate this now (2, 32).

Consistent with previous reports in other systems (38, 40), we observed some leaky DiCre activity, and we documented excision in the dispensable TK gene in the absence of rapamycin. We did not conduct rigorous serial passage experiments, but it is likely that the floxed segment is successively lost over time. In contrast, we did not observe such loss for the floxed AP2-F parasites in the absence of rapamycin induction. While there are several potential explanations for this difference, the most likely reason may be that excision of an essential gene will result in loss of those parasites from the population, thus providing strong purifying counterselection.

When rapamycin was added to infected cultures, we observed inducible Cre activity in both strains and with similar kinetics. The number of parasites that express the AP2-F protein was reduced by 87% over 48 h. Loss of AP2-F did not significantly affect the growth and asexual amplification of parasites in culture (or in mice), consistent with its role, being specifically restricted to the biology of the female gamete. Loss of the protein also did not impact commitment to female fate or development, as numbers of female gametes were indistinguishable between treated and control experiments. However, when we induced loss of AP2-F during infection in mice, we observed reduced shedding of oocysts that was not noted in a control strain. Overall, we conclude that AP2-F, and the genes under its control, are essential for the completion of the C. parvum life cycle and required for the ability of the female gamete to undergo fertilization or postfertilization development, leading to the formation and release of new infectious oocysts. Future studies using electron microscopy may reveal more subtle changes in the ultrastructure of the female gamete that were not noted in our experiments.

What may be the molecular nature of that step? Sequencing analyses of AP2-F in culture revealed reduced transcription of a focused set of genes, with a by-and-large exclusively female expression pattern. Among them were meiosis-associated proteins, including NIMA kinase. Plasmodium berghei homologs of this protein were shown to be required for meiosis and completion of the mosquito portion of the malaria life cycle (49, 50). However, most prominent among the transcripts lost were those that encode most of the components of the crystalloid body. We recently defined the composition of this organelle, along with that of many other sporozoite compartments, in a proteomic experiment that used the hyperplexed localization of organelle proteins by isotope tagging (Hyper-LOPIT) approach (51; A. Guérin and B. Striepen). We tested for gene set enrichment among the genes impacted by loss of AP2-F and found crystalloid body, but not oocyst wall proteins, to be highly enriched (Fig. S6; *P* < 0.001; false-discovery rate [FDR], 0). The crystalloid body is a multivesicular organelle containing an ordered array of spherical vesicles of 25 to 60 nm in diameter and is found at the basal end of *Cryptosporidium* sporozoites but absent from merozoites (52, 53). Crystalloid bodies have been observed in other apicomplexans (53), and the structure is best characterized in *Plasmodium* species, where it is found in the postfertilization ookinete and early oocyst stages (54). The molecular components of the crystalloid body are proteins that carry signal peptides, and feature complex interaction domains, including the CCp family of proteins, which share similarity with the *Limulus* coagulation factor C, or Pleckstrin homology domain proteins, and several members have homologs in both *Plasmodium* and *Cryptosporidium* (55, 56). Importantly, mutants in these crystalloid body proteins, as well as related gametocyte proteins in *Plasmodium*, were demonstrated to suffer aberrant oocyst and/or sporozoite formation leading to loss of transmission from insect to mammal (55, 57). This is an intriguing parallel and is consistent with the phenotype observed here; however, further work is required to fully elaborate and test this model, as we currently lack antibody reagents in C. parvum crystalloid body proteins to directly test such a role.

10.1128/mbio.03261-22.6 FIG S6 Gene set enrichment analyses of female gene signatures upon the loss of AP2-F. Analyses were conducted using GSEA v.4.3.2 to show enrichment in the untreated control (reflecting loss upon rapamycin treatment). (A and B) Plots shown use the Hyper-LOPIT-defined (A. Guérin and B. Striepen, unpublished) C. parvum gene sets for the crystalloid body (A) and oocyst wall (B) (as a female-specific control). Download FIG S6, TIF file, 0.9 MB. Copyright © 2023 Tandel et al. 2023 Tandel et al. https://creativecommons.org/licenses/by/4.0/ This content is distributed under the terms of the Creative Commons Attribution 4.0 International license.

AP2 DNA binding proteins have been identified as regulators of stage differentiation, including female development and ookinete formation in *Plasmodium* (23, 24, 26, 58, 59), and we show in this study that they also control key steps in *Cryptosporidium* male and female biology. The simplicity of its single host life cycle makes C. parvum a powerful model to dissect the fundamental biology of apicomplexan development. Conditional gene ablation as developed here, and induced protein destabilization as described in a recent study by Choudhary and colleagues (37), now open the door to rigorous genetic analysis of this fascinating process.

## MATERIALS AND METHODS

### Mice.

IFN-γ^–/–^ mice (stock no. 002287) were purchased from Jackson Laboratory and bred in-house. Mice of both sexes were used (typically ranging from 6 to 8 weeks in age). Mice were treated with antibiotics, infected, and handled as detailed in reference 60.

### Cells.

HCT-8 cells were purchased from ATCC (CCL-224TM) and maintained in RPMI 1640 medium (Sigma-Aldrich) supplemented with 10% Cosmic calf serum (HyClone), 1% penicillin-streptomycin (Gibco), 1% l-glutamine (Gibco), and 1× amphotericin B (GoldBio). For HCT-8 infection medium, serum concentrations were reduced to 1%.

### Reagents and drugs.

If not indicated specifically, reagents were purchased from Sigma-Aldrich. Rapamycin (LC Laboratories) was dissolved in 95% ethanol at a stock concentration of 50 mg/mL. For *in vivo* studies, rapamycin was further diluted in water. Mice were weighed and treated daily by oral gavage with 100 μL of drug solution adjusted to deliver 10 mg/kg body weight (note that the ethanol concentration must not exceed 5% and was 2.5% in most of our experiments). A vehicle control was established from 95% ethanol by equivalent dilution.

### Antibodies and stains.

Primary antibodies used in this study include rat anti-HA (Roche clone 3F10), VVL-FITC (1:1,000, Vector FL1231); DMC1 antibody (1:10, a kind gift of Christopher Huston, University of Vermont [15]), rabbit anti-TrpB (31) and mouse anti-alpha tubulin (a kind gift of Jacek Gaertig, University of Georgia), both used at 1:300; and secondary antibodies carrying a variety of Alexa Fluors (Abcam) at 1:1,000.

### Plasmid construction.

The DiCre cassette was built using a P. falciparum construct (40) kindly shared by Tobias Spielmann (Bernhard Nocht Insitut Hamburg, Germany). Guide oligonucleotides (Sigma-Aldrich) were introduced into the C. parvum Cas9/U6 plasmid16 by restriction cloning. See reference 61 for a detailed discussion of guide design for C. parvum. Transfection plasmids were constructed by Gibson assembly using NEB Gibson assembly master mix (New England Biolabs). File S2 provides the sequences of all primers used in this study. Note that constructing plasmids that contained both the DiCre expression cassette and the floxed segment often resulted in premature excision of the floxed segment in bacteria due to Cre activity. We thus engineered the two pieces independently and then generated our linear targeting constructs by fusing PCR amplicons containing the Dicre cassette and the floxed segments, respectively, by Gibson assembly (we recommend a 15- to 20-bp overlap between amplicons for ensuring fusion). The final assembled product was then PCR amplified using terminal primers and used in transfection experiments.

### Parasite strains.

We generated and purified transgenic parasite lines using Cryptosporidium parvum Iowa II strain oocysts (Bunchgrass Farms, Deary, ID) following previously detailed protocols (31, 60, 61).

### Nanoluciferase assay.

The Nano-Glo luciferase assay system (Promega) and Glomax 3000 (Promega) were used to measure luciferase activity in parasites, following the manufacturer’s protocol unless otherwise noted. For fecal samples, 19 to 21 mg of feces was processed as described previously in reference 61. For *in vitro* cultures, monolayers were directly resuspended in the NanoGlo substrate solution, and luminescence measured. For intestinal samples, gut punches were weighed and lysed in fecal lysis buffer (61) by vortexing for 10 min with glass beads. Samples were mixed 1:1 with NanoGlo substrate solution and assayed in triplicate.

### Transient transfection assay for engineering the DiCre conditional system.

We followed the protocols for transient transfection of parasites as described (31). Briefly, 1 × 10^7^ C. parvum oocysts (Bunchgrass Farms, Deary, ID) were excysted and transfected with 10 μg each of DiCre and floxed plasmids using an Amaxa 4D electroporator (Lonza). The transfected parasites were diluted in HCT-8 infection medium and divided equally to infect three 24-well plates of subconfluent HCT-8 cells. Nanoluciferase activity was measured 48 h after infection.

### PCR assay for DiCre activation in stable transgenics.

HCT-8 cells were infected with 50,000 oocysts in HCT-8 infection medium with and without 100 nM rapamycin. After different times of infection, DNA was isolated from the cells according to the quick-start protocol from the DNeasy blood and tissue kit (Qiagen). The PCR primers used are listed in supplemental Data Set S2.

### Immunofluorescence assay.

HCT-8 cells were grown on coverslips in either 24- or 96-well plates and 20,000 of prepared oocysts in HCT-8 infection medium, respectively. For DiCre activation experiments, parasites were also grown in the presence of 100 nM rapamycin. Cells were fixed with 4% paraformaldehyde (Electron Microscopy Science) in phosphate-buffered saline (PBS) and then permeabilized with PBS containing 0.25% Triton X-100. The cells were blocked with 3% bovine serum albumin (BSA, Sigma-Aldrich) solution, followed by incubation with primary antibodies. Cells were washed with PBS and then incubated with appropriate fluorophore-conjugated secondary antibodies and counterstained with DAPI (4′,6-diamidino-2-phenylindole; Thermo Fisher). Coverslips were then mounted on glass slides with Fluoro-Gel (Electron Microscopy Science) mounting medium. Superresolution structured illumination microscopy was conducted using a Carl Zeiss Elyra (UGA Biomedical Microscopy Core) or a GE OMX (PennVet Imaging Core) microscope. Wide-field microscopy was performed using a Leica LAS X microscope (PennVet Imaging Core), and images were processed and analyzed using Carl Zeiss ZEN v.2.3 SP1, GE Softworx, and NIH ImageJ software.

### EdU labeling to measure thymidine kinase activity.

HCT-8 cells were infected with 100,000 oocysts of the TK Flox Dicre strain. EdU (Thermo Fisher) was added to cultures 36 h after infection to a final concentration of 10 μM, and cells were fixed 12 h later. A Click-iT EdU Alexa-Fluor 594 kit (Thermo Fisher Scientific) was used to label incorporated EdU. Parasites were counterstained with anti-HA antibody and fluorescein-conjugated *Vicia villosa* lectin (Vector Laboratories).

### Rapamycin induction *in vivo*.

Female IFN-γ^−/−^ mice (4 to 6 weeks old) were treated with 10 mg/kg of rapamycin (LC Laboratories) via oral gavage daily, starting 2 days prior to infection and continuing 5 days postinfection. Mice were sex and age matched between treatment groups. Following 2 days of pretreatment with rapamycin, mice were gavaged with 10,000 oocysts in PBS. Bulk fecal samples were collected, and intestinal punches were harvested on day 6. Punches were stored at –80°C prior to processing for nanoluciferase measurement.

### RNA sequencing.

HCT-8 cells were infected with 100,000 oocysts of wild-type (nontransgenic) C. parvum or AP2-F DiCre strains with or without 100 nM rapamycin. Cells were lysed and RNA was extracted using the RNeasy minikit (Qiagen, Hilden, Germany) according to the manufacturer’s protocol. The Illumina stranded mRNA prep ligation kit (20040534) was used for cDNA generation and library preparation. Total RNA and libraries were quality controlled and quantified using the TapeStation 4200 (Agilent Technologies, Santa Clara, CA) and Qubit 3 (Thermo Fisher Scientific, Waltham, MA) instruments. Samples were pooled, and single-end reads were generated on an Illumina NextSeq 500 sequencer.

### RNA sequencing analysis.

Raw reads were mapped to the C. parvum Iowa II reference (VEuPathDB, release 50) using Kallisto v.0.45.0 (62). All subsequent analyses were carried out using the statistical computing environment R v.4.0.3 in RStudio v.1.3.1093 and Bioconductor. In brief, transcript-level quantification data were summarized to genes using the tximport package, and data were normalized using the trimmed mean of M values method, which was implemented in EdgeR (63). Only genes with more than 10 counts per million in at least 3 samples were used for analysis. Precision weights were applied to each gene based on the mean-variance relationship using the VOOM function in limma (64). Linear modeling and Bayesian statistics carried out in limma were used to identify differentially expressed genes with an FDR-adjusted *P* value of ≤0.01 and an absolute log_2_-transformed fold change of ≥1 after adjustment with Benjamini-Hochberg correction. Genes were identified as oocyst wall and crystalloid body based on analyses derived from the C. parvum Hyper-LOPIT proteomic data set (A Guérin, K Strelau, K Barylyuk, B Wallbank, L Berry, OM Crook, KS Lilley, RF Waller, B Striepen, submitted for publication). Gene set enrichment analysis was carried out using Gene Set Enrichment Analysis (GSEA) software v.4.3.2 (65). Oocyst wall and crystalloid body signatures obtained from the Hyper-LOPIT data set were used for GSEA with 1,000 permutations, a weighted enrichment statistic, and the Diff_of_Classes metric for ranking genes. All other parameters were kept at the default settings. All code is found in supplemental Data Set S3.

### Statistical methods.

GraphPad PRISM was used for all statistical analyses. When measuring the difference between the two populations, we used a standard Student’s *t* test. No statistical tests were used to predetermine sample size, and no animals were excluded from the results. Analysis of variance (ANOVA) was used to compare the means of multiple groups, followed by Tukey’s *post hoc* test for pairwise comparisons.

### Animal ethics statement.

All the protocols for animal experimentation were approved by the Institutional Animal Care and Use Committee of the University of Georgia (protocol A2016 01-028-Y1-A4) and/or the Institutional Animal Care and Use Committee of the University of Pennsylvania (protocol number 806292). No statistical tests were used to predetermine the sample size of mice used for experiments. Mice were not randomized, and investigators were not blinded before any of the experiments.

### Data availability.

The RNA sequencing data generated in this study are available from the GEO database repository under accession number GSE216844. All code used in these analyses is available in Data Set S3 and on GitHub (https://github.com/katelyn-walzer/Ablation_of_AP2-F_Manuscript).

10.1128/mbio.03261-22.8 DATA SET S1 Differentially expressed genes. Multitab Excel file containing differentially expressed genes between all samples. The first tab contains all differentially expressed genes, and the other tabs contain comparisons between individual samples. AP2-F is highlighted in red. Crystalloid body proteins are highlighted in magenta. Expression data are normalized counts per million and are log2 transformed. Download Data Set S1, XLS file, 0.05 MB. Copyright © 2023 Tandel et al. 2023 Tandel et al. https://creativecommons.org/licenses/by/4.0/ This content is distributed under the terms of the Creative Commons Attribution 4.0 International license.

10.1128/mbio.03261-22.9 DATA SET S2 Table of all oligonucleotide primers used in this study. Download Data Set S2, PDF file, 0.03 MB. Copyright © 2023 Tandel et al. 2023 Tandel et al. https://creativecommons.org/licenses/by/4.0/ This content is distributed under the terms of the Creative Commons Attribution 4.0 International license.

10.1128/mbio.03261-22.10 DATA SET S3 Detail of the computer code used in the course of the RNA sequence analysis used in this study. Download Data Set S3, HTML file, 2.4 MB. Copyright © 2023 Tandel et al. 2023 Tandel et al. https://creativecommons.org/licenses/by/4.0/ This content is distributed under the terms of the Creative Commons Attribution 4.0 International license.
