# Supplementary figures and images for: Genetic Ablation of a Female-Specific Apetala 2 Transcription Factor Blocks Oocyst Shedding in Cryptosporidium parvum
Source: mBio. 2023 Feb 14;14(2):e03261-22. doi: 10.1128/mbio.03261-22 (PMC10233709; doi:10.1128/mbio.03261-22)

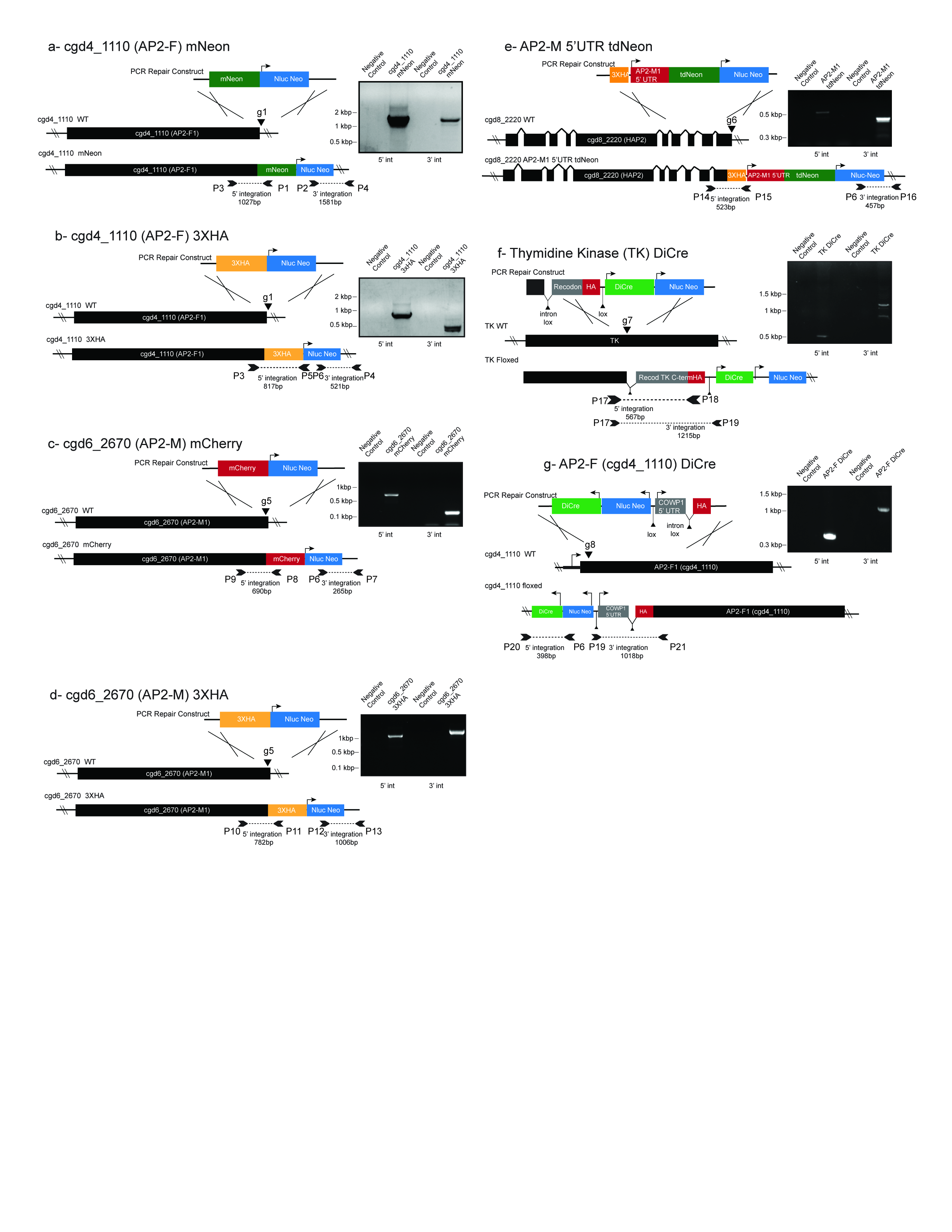

Supplement: FIG S1 [file mbio.03261-22-s0001.tif]

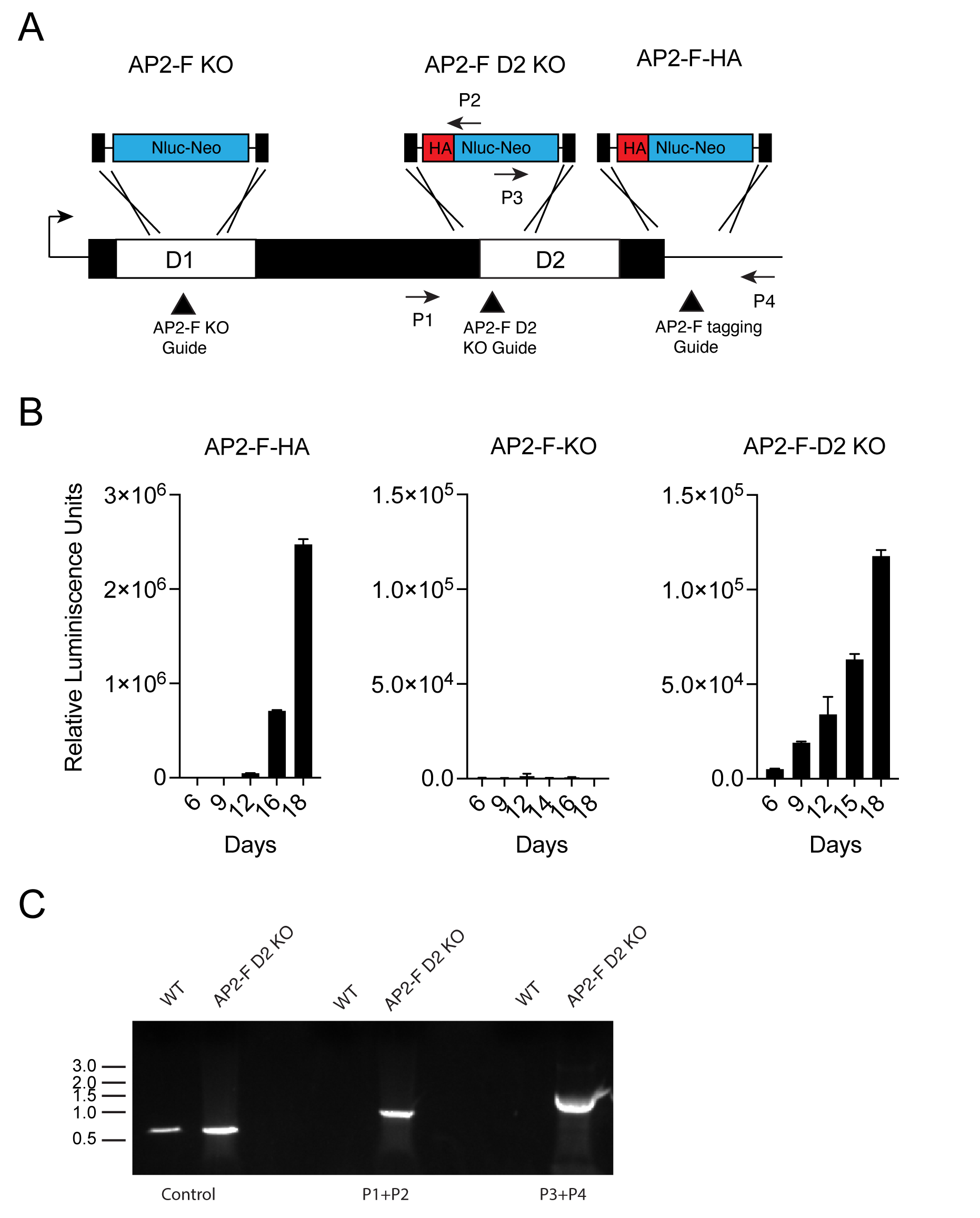

Supplement: FIG S2 [file mbio.03261-22-s0002.tif]

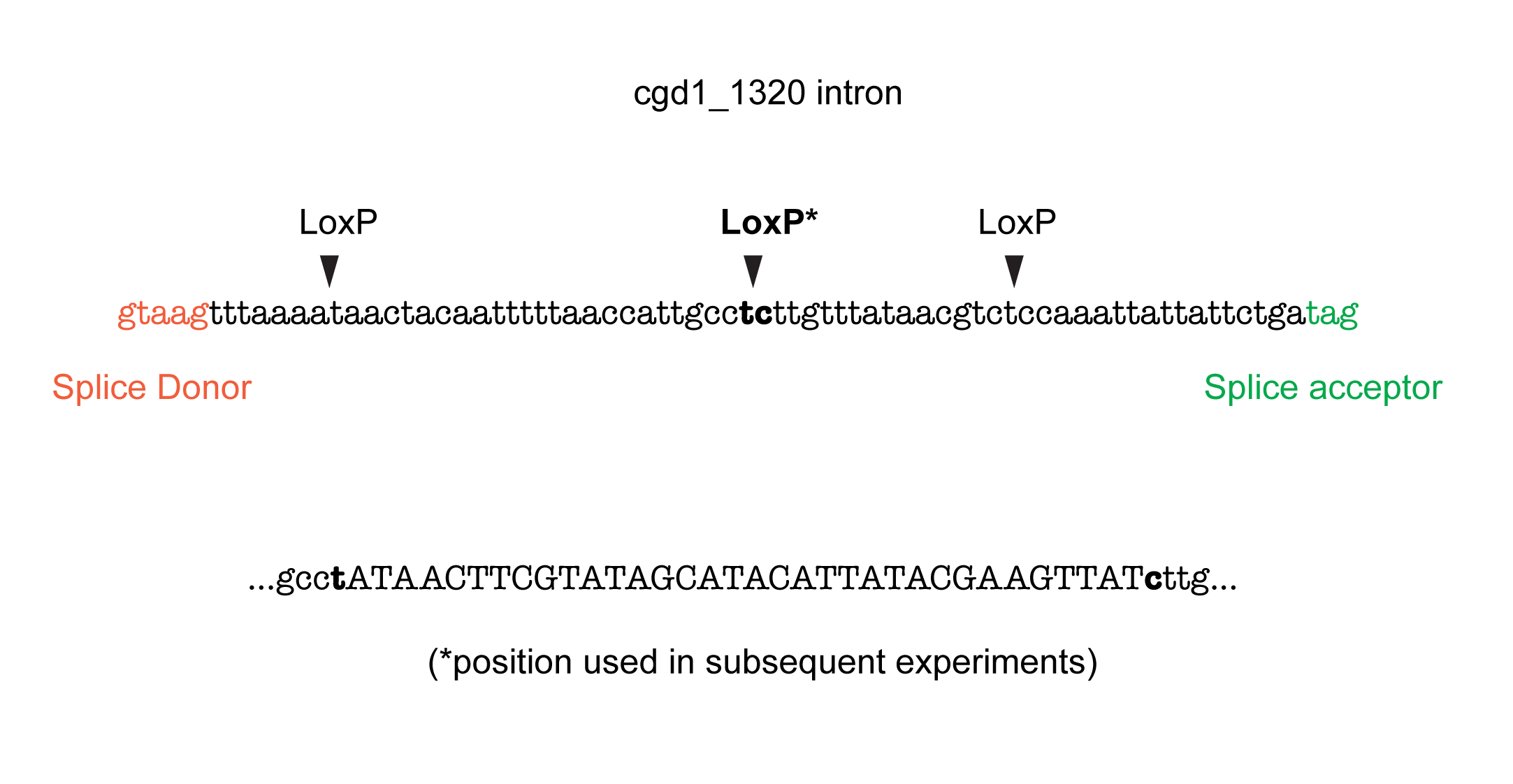

Supplement: FIG S3 [file mbio.03261-22-s0003.tif]

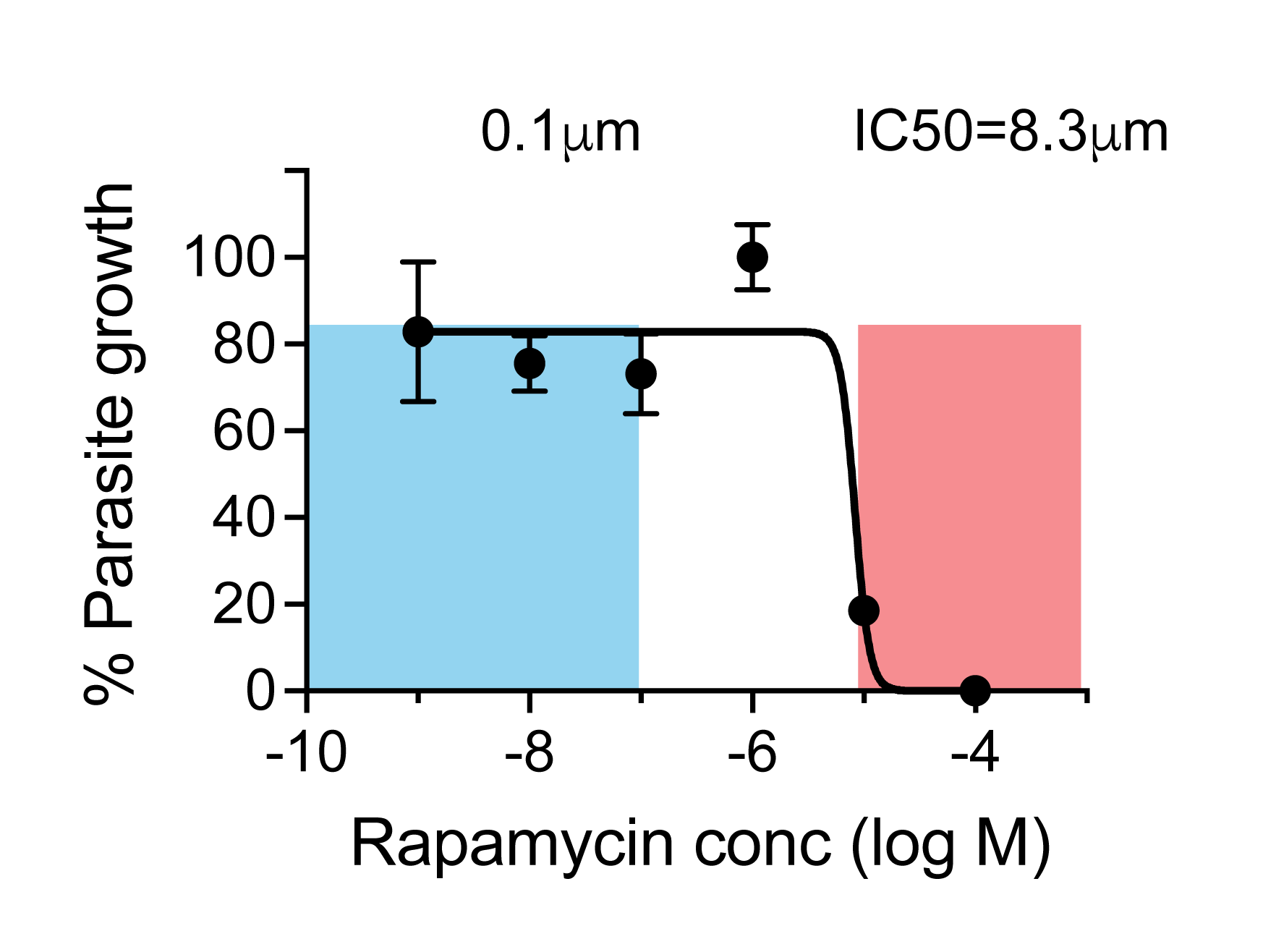

Supplement: FIG S4 [file mbio.03261-22-s0004.tif]

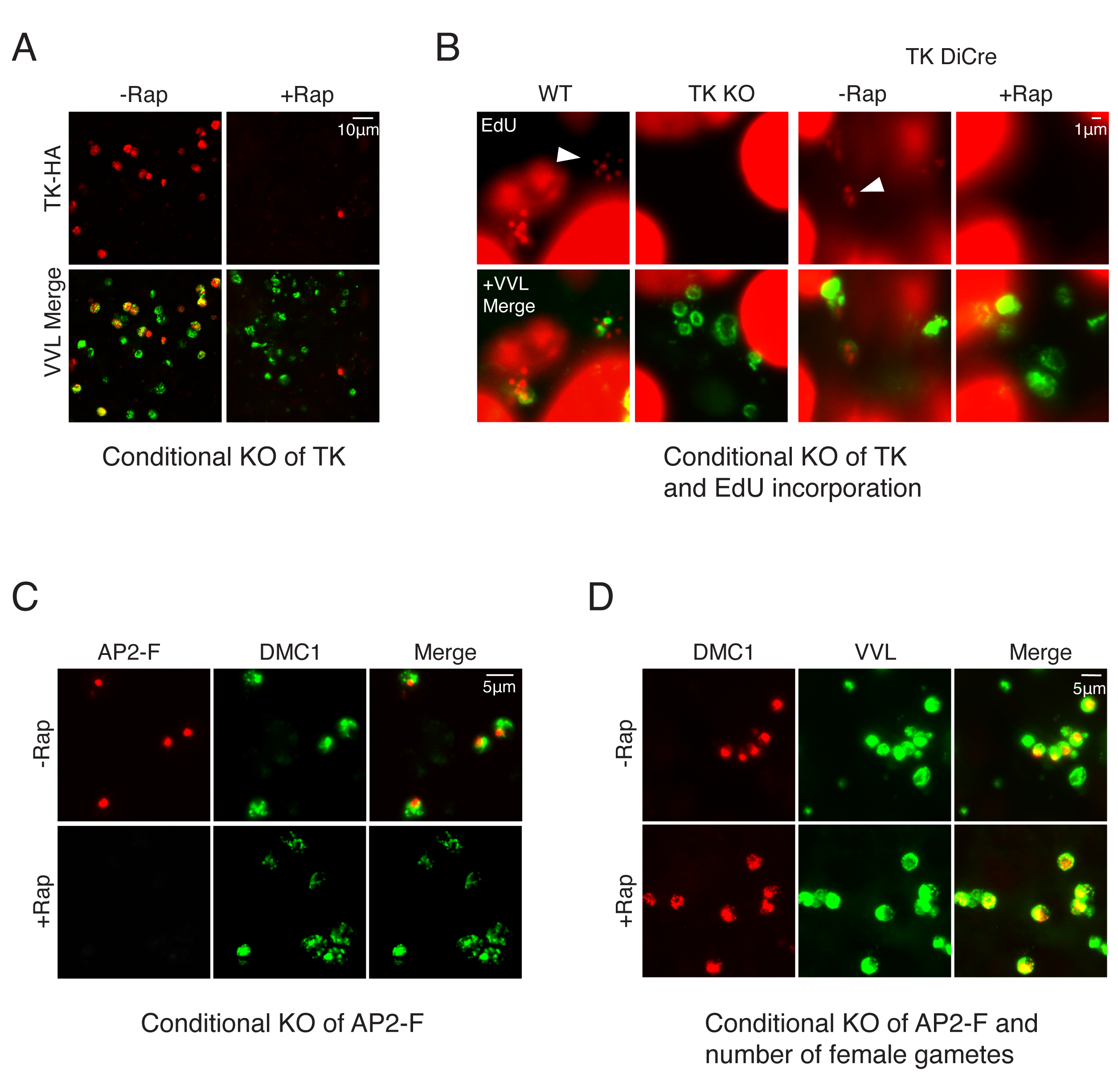

Supplement: FIG S5 [file mbio.03261-22-s0005.tif]

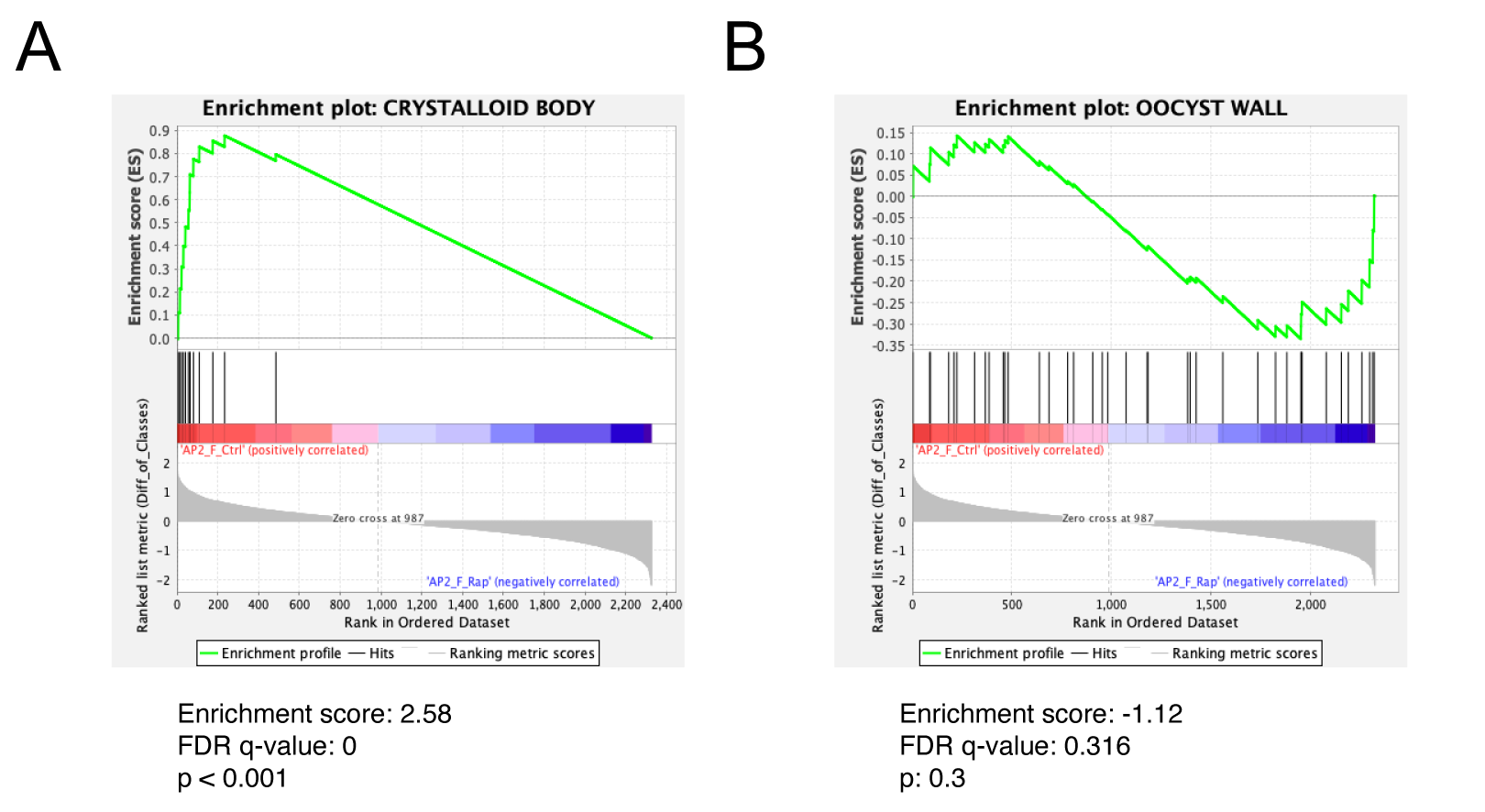

Supplement: FIG S6 [file mbio.03261-22-s0006.tif]
