# Supplementary material for: Genetic Ablation of a Female-Specific Apetala 2 Transcription Factor Blocks Oocyst Shedding in Cryptosporidium parvum
Source: mBio. 2023 Feb 14;14(2):e03261-22. doi: 10.1128/mbio.03261-22 (PMC10233709; doi:10.1128/mbio.03261-22)
Supplement: DATA SET S2 [file mbio.03261-22-s0008.pdf]

Supplementary File S2: Primers used in this study

Guides and primer name	5'-3' sequence
AP2-F1 C-terminus tagging guide (g1)	gtaatttaataaaatgttta
AP2-F1 KO guide 1(g2)	gagaagagcagaacatcaaag
AP2-F1 KO guide 2 (g3)	gtcaaagatggattgctcag
AP2-F1 D2 KO guide (g4)	gaagactagagatcaagaat
AP2-M1 C-terminus tagging guide (g5)	gattatatttggtgatttcaa
HAP2 C-terminus tagging guide (g6)	gagccaagaaagtttaagtca
Thymidine kinase floxing guide (g7)	gaagtaaatacttattagca
AP2-F N-terminus floxing guide (g8)	gatggaggaaaaattagaaac
P1	aaataaagtaaagtttatcgacctaagatactaaatga
P2	ctagcatgattgaacaagatggtttacacgctgg
P3	ggaagacggaggagattgtctcaatact
P4	gcaatgactacatgtactttcccgctc
P5	ctttggatcggagttacggacac
P6	gctcccgattctcaacgtatcgccttctatc
P7	taggacaatgattgagaagatgacttcaaaca
P8	cagcccatggtcttcttctgcatt
P9	tatgcaacccaagaatacatggaaaa
P10	gccatcagaagatggccactatacaatccatt
P11	cttcgagtgtgaagaccatgtc
P12	atgattgaacaagatggtttacacg
P13	atcgttggccctagaactcttaaaactactctcg
P14	caataatttctgatgtagtagtgaatcaaact
P15	aaataaagtaaagtttatcgacctaagatactaaatga
P16	tgaaaataatgaaatcgtattggtatccc
P17	agcaatgaatgctggaaaatcaacg
P18	ccgccttagaaattgtattcttcac
P19	gtttaacgaataactgtttaacgaataactttaac
P20	gagcagagtttgtgaagaattattttgaaaacaaga
P21	gagattggacttgttgaattttactattaccaac
P22	atctggaaaaaattgacccattaagagaagaaatg
P23	atgacgcaatatagaactaagtgtgtg
