# Supplementary material for: Genetic Ablation of a Female-Specific Apetala 2 Transcription Factor Blocks Oocyst Shedding in Cryptosporidium parvum
Source: mBio. 2023 Feb 14;14(2):e03261-22. doi: 10.1128/mbio.03261-22 (PMC10233709; doi:10.1128/mbio.03261-22)
Supplement: DATA SET S3 [file mbio.03261-22-s0009.html]

Genetic ablation of a female specific Apetala 2 transcription factor blocks oocyst shedding in Cryptosporidium parvum


Code 

- Show All Code
- Hide All Code

# Genetic ablation of a female specific Apetala 2 transcription factor blocks oocyst shedding in *Cryptosporidium parvum*

#### *Jayesh Tandel, Katelyn A. Walzer, Jessica H. Byerly, Brittain Pinkston, Daniel P. Beiting, and Boris Striepen*

#### *2022-11-22*

# 1 Introduction

This reproducible and dynamic report was created using Rmarkdown and the Knitr package, and summarizes the basic code and outputs (plots, tables, etc) produced for this manuscript.

---

# 2 Background

The apicomplexan parasite *Cryptosporidium* is a leading global cause of diarrheal disease, and the infection poses a particularly grave threat to young children, and those with weakened immune function. Infection occurs by ingestion of meiotic spores called oocysts, and transmission relies on fecal shedding of new oocysts. The entire lifecycle thus occurs in a single host and features asexual as well as sexual forms of replication. Here we identify and locus tag two Apetala 2-type (AP2) transcription factors and demonstrate that they are exclusively expressed in male and female gametes, respectively. To enable functional studies of essential genes in *C. parvum* we develop and validate a small molecule inducible gene excision system, which we apply to the female factor AP2-F to achieve conditional gene knock out. Analyzing this mutant, we find the factor to be dispensable for asexual growth and early female fate determination in vitro, but to be required for oocyst shedding in infected animals in vivo. Transcriptional analyses conducted in the presence or absence of AP2-F reveal that the factor controls the transcription of genes encoding crystalloid body proteins. These proteins are exclusively expressed in female gametes and in *C. parvum* the organelle is restricted to sporozoites.

The code below shows how the raw data was preprocessed, mapped, and analyzed to identify the gene expression changes that occur upon loss of AP2-F.

---

# 3 Reproducibility and accessibility

In order to reproduce all steps listed below, including QC of raw reads and read mapping, raw fastq files will need to be downloaded from the Gene Expression Omnibus, under accession GSE216844. Prealigned data and all code used in this analysis, including the Rmarkdown document used to compile this supplementary code file, are all available on GitHub here. Once this GitHub repo has been downloaded, navigate to /Ablation\_of\_AP2-F\_Manuscript/ANALYSIS/code to find the Rmarkdown document as well as an RProject file. This should be your working directory for executing code.

---

# 4 R packages used for this analysis

A variety of R packages was used for this analysis. All graphics and data wrangling were handled using the tidyverse suite of packages. All packages used are available from the Comprehensive R Archive Network (CRAN), Bioconductor.org, or Github.

```
library(tidyverse) 
library(tximport)
library(biomaRt)
library(hrbrthemes) 
library(RColorBrewer) 
library(reshape2)
library(genefilter) 
library(edgeR) 
library(matrixStats)
library(DT) 
library(gt) 
library(plotly) 
library(skimr)
library(limma)
```

---

# 5 Read mapping

## 5.1 Aligning raw reads with Kallisto

Quality control of raw reads was carried out using fastqc. Raw reads were mapped to the version 50 *Cryptosporidium parvum* Iowa II reference transcriptome available on VEuPathDB using Kallisto, version 0.45.0.

```
#Kallisto make new reference using CryptoDB version 50
#Use annotated transcripts file
kallisto index -i CryptoDB-50_CparvumIowaII_AnnotatedTranscripts.index CryptoDB-50_CparvumIowaII_AnnotatedTranscripts.fasta


#Run fastqc
fastqc WTControl1-Jayesh-Katie_S1_mergedLanes_R1_001.fastq.gz -t 24 -o /data/striepenlab/Katelyn_Walzer_data/Jayesh_manuscript/AP2_F_Knockout/fastqc
fastqc WTControl2-Jayesh-Katie_S2_mergedLanes_R1_001.fastq.gz -t 24 -o /data/striepenlab/Katelyn_Walzer_data/Jayesh_manuscript/AP2_F_Knockout/fastqc
fastqc WTControl3-Jayesh-Katie_S3_mergedLanes_R1_001.fastq.gz -t 24 -o /data/striepenlab/Katelyn_Walzer_data/Jayesh_manuscript/AP2_F_Knockout/fastqc
fastqc WTRap1-Jayesh-Katie_S4_mergedLanes_R1_001.fastq.gz -t 24 -o /data/striepenlab/Katelyn_Walzer_data/Jayesh_manuscript/AP2_F_Knockout/fastqc
fastqc WTRap2-Jayesh-Katie_S5_mergedLanes_R1_001.fastq.gz -t 24 -o /data/striepenlab/Katelyn_Walzer_data/Jayesh_manuscript/AP2_F_Knockout/fastqc
fastqc WTRap3-Jayesh-Katie_S6_mergedLanes_R1_001.fastq.gz -t 24 -o /data/striepenlab/Katelyn_Walzer_data/Jayesh_manuscript/AP2_F_Knockout/fastqc
fastqc AP2-FControl1-Jayesh-Katie_S7_mergedLanes_R1_001.fastq.gz -t 24 -o /data/striepenlab/Katelyn_Walzer_data/Jayesh_manuscript/AP2_F_Knockout/fastqc
fastqc AP2-FControl2-Jayesh-Katie_S8_mergedLanes_R1_001.fastq.gz -t 24 -o /data/striepenlab/Katelyn_Walzer_data/Jayesh_manuscript/AP2_F_Knockout/fastqc
fastqc AP2-FControl3-Jayesh-Katie_S9_mergedLanes_R1_001.fastq.gz -t 24 -o /data/striepenlab/Katelyn_Walzer_data/Jayesh_manuscript/AP2_F_Knockout/fastqc
fastqc AP2-FRap1-Jayesh-Katie_S10_mergedLanes_R1_001.fastq.gz -t 24 -o /data/striepenlab/Katelyn_Walzer_data/Jayesh_manuscript/AP2_F_Knockout/fastqc
fastqc AP2-FRap2-Jayesh-Katie_S11_mergedLanes_R1_001.fastq.gz -t 24 -o /data/striepenlab/Katelyn_Walzer_data/Jayesh_manuscript/AP2_F_Knockout/fastqc
fastqc AP2-FRap3-Jayesh-Katie_S12_mergedLanes_R1_001.fastq.gz -t 24 -o /data/striepenlab/Katelyn_Walzer_data/Jayesh_manuscript/AP2_F_Knockout/fastqc


#Run alignment with kallisto to CryptoDB version 50
#Average length of fragment is 300 bp from TapeStation
#Decrease standard deviation to 30
kallisto quant -i CryptoDB-50_CparvumIowaII_AnnotatedTranscripts.index -o WT_Ctrl_rep_1 -t 24 -b 60 --single -l 300 -s 30 WTControl1-Jayesh-Katie_S1_mergedLanes_R1_001.fastq.gz
kallisto quant -i CryptoDB-50_CparvumIowaII_AnnotatedTranscripts.index -o WT_Ctrl_rep_2 -t 24 -b 60 --single -l 300 -s 30 WTControl2-Jayesh-Katie_S2_mergedLanes_R1_001.fastq.gz
kallisto quant -i CryptoDB-50_CparvumIowaII_AnnotatedTranscripts.index -o WT_Ctrl_rep_3 -t 24 -b 60 --single -l 300 -s 30 WTControl3-Jayesh-Katie_S3_mergedLanes_R1_001.fastq.gz
kallisto quant -i CryptoDB-50_CparvumIowaII_AnnotatedTranscripts.index -o WT_Rap_rep_1 -t 24 -b 60 --single -l 300 -s 30 WTRap1-Jayesh-Katie_S4_mergedLanes_R1_001.fastq.gz
kallisto quant -i CryptoDB-50_CparvumIowaII_AnnotatedTranscripts.index -o WT_Rap_rep_2 -t 24 -b 60 --single -l 300 -s 30 WTRap2-Jayesh-Katie_S5_mergedLanes_R1_001.fastq.gz
kallisto quant -i CryptoDB-50_CparvumIowaII_AnnotatedTranscripts.index -o WT_Rap_rep_3 -t 24 -b 60 --single -l 300 -s 30 WTRap3-Jayesh-Katie_S6_mergedLanes_R1_001.fastq.gz
kallisto quant -i CryptoDB-50_CparvumIowaII_AnnotatedTranscripts.index -o AP2_F_Ctrl_rep_1 -t 24 -b 60 --single -l 300 -s 30 AP2-FControl1-Jayesh-Katie_S7_mergedLanes_R1_001.fastq.gz
kallisto quant -i CryptoDB-50_CparvumIowaII_AnnotatedTranscripts.index -o AP2_F_Ctrl_rep_2 -t 24 -b 60 --single -l 300 -s 30 AP2-FControl2-Jayesh-Katie_S8_mergedLanes_R1_001.fastq.gz
kallisto quant -i CryptoDB-50_CparvumIowaII_AnnotatedTranscripts.index -o AP2_F_Ctrl_rep_3 -t 24 -b 60 --single -l 300 -s 30 AP2-FControl3-Jayesh-Katie_S9_mergedLanes_R1_001.fastq.gz
kallisto quant -i CryptoDB-50_CparvumIowaII_AnnotatedTranscripts.index -o AP2_F_Rap_rep_1 -t 24 -b 60 --single -l 300 -s 30 AP2-FRap1-Jayesh-Katie_S10_mergedLanes_R1_001.fastq.gz
kallisto quant -i CryptoDB-50_CparvumIowaII_AnnotatedTranscripts.index -o AP2_F_Rap_rep_2 -t 24 -b 60 --single -l 300 -s 30 AP2-FRap2-Jayesh-Katie_S11_mergedLanes_R1_001.fastq.gz
kallisto quant -i CryptoDB-50_CparvumIowaII_AnnotatedTranscripts.index -o AP2_F_Rap_rep_3 -t 24 -b 60 --single -l 300 -s 30 AP2-FRap3-Jayesh-Katie_S12_mergedLanes_R1_001.fastq.gz
```

---

## 5.2 Importing count data into R

After read mapping with Kallisto, TxImport was used to read kallisto outputs into the R environment.

```
# read in study design ----
targets <- read_tsv("Study_Design_AP2_F_DiCre_Feb_2021.txt")

# create file paths to the abundance files generated by Kallisto using the 'file.path' function
path <- file.path(targets$sample, "abundance.h5")

# check to make sure this path is correct by seeing if the files exist
all(file.exists(path))
```

```
## [1] TRUE
```

```
# use dplyr to modify study design to include these file paths as a new column.
targets <- mutate(targets, path)

# import Kallisto transcript counts into R using Tximport ----
Txi_gene <- tximport(path, 
                     type = "kallisto", 
                     txOut = TRUE,
                     countsFromAbundance = "lengthScaledTPM")

myCPM <- as_tibble(Txi_gene$abundance, rownames = "geneSymbol") # counts after adjusting for transcript length
myCounts <- as_tibble(Txi_gene$counts, rownames = "geneSymbol") # counts per million (CPM)
```

---

# 6 Identification of differentially expressed genes upon rapamycin treatment and AP2-F knockout

## 6.1 Filtering and normalization

```
# Identify variables of interest in study design file ----
groups1 <- targets$strain_condition
groups1 <- factor(groups1)
sampleLabels <- targets$sample

# Examine data up to this point ----
myCPM <- Txi_gene$abundance
myCounts <- Txi_gene$counts

# graph both matrices 
colSums(myCPM)
```

```
##  [1] 1e+06 1e+06 1e+06 1e+06 1e+06 1e+06 1e+06 1e+06 1e+06 1e+06 1e+06 1e+06
```

```
colSums(myCounts)
```

```
##  [1] 239764 238656 365374 215298 227140 139728 550203 108982 203411 164752
## [11] 157243 172522
```

```
# Take a look at the heteroskedasticity of the data ----
# first, calculate row means and standard deviations for each transcript or gene 
# and add these to the data matrix
myCPM.stats <- transform(myCPM, 
                         SD=rowSds(myCPM), 
                         AVG=rowMeans(myCPM),
                         MED=rowMedians(myCPM)
)


# produce a scatter plot of the transformed data
ggplot(myCPM.stats, aes(x=SD, y=MED)) +
  geom_point(shape=16, size=2)
```

```
# Make a DGElist from the counts, and plot ----
myDGEList <- DGEList(Txi_gene$counts)


save(myDGEList, file = "myDGEList")


# use the 'cpm' function from EdgeR to get counts per million
cpm <- cpm(myDGEList) 
log2.cpm <- cpm(myDGEList, log=TRUE)


# Take a look at the distribution of the Log2 CPM
nsamples <- ncol(log2.cpm)
# now select colors from a single palette
myColors <- brewer.pal(nsamples, "Paired")

# 'coerce' the data matrix to a dataframe to use tidyverse tools on it
log2.cpm.df <- as_tibble(log2.cpm)

colnames(log2.cpm.df) <- sampleLabels

# use the reshape2 package to 'melt' dataframe (from wide to tall)
log2.cpm.df.melt <- melt(log2.cpm.df)


ggplot(log2.cpm.df.melt, aes(x=variable, y=value, fill=variable)) +
  geom_violin(trim = FALSE, show.legend = FALSE) +
  stat_summary(fun.y = "median", geom = "point", shape = 124, size = 6, color = "black", show.legend = FALSE) +
  labs(y="log2 expression", x = "sample",
       title="Log2 Counts per Million (CPM)",
       subtitle="unfiltered, non-normalized",
       caption=paste0("produced on ", Sys.time())) + 
  coord_flip()
```

```
# Filter the data ----
#first, take a look at how many genes or transcripts have no read counts at all
table(rowSums(myDGEList$counts==0)==12)
```

```
## 
## FALSE  TRUE 
##  3808   215
```

```
# set some cut-off to get rid of genes/transcripts with low counts
keepers <- rowSums(cpm>10)>=3 #last number is replicates, min replicates, at least expressed ten times
myDGEList.filtered <- myDGEList[keepers,]
dim(myDGEList.filtered)
```

```
## [1] 2327   12
```

```
log2.cpm.filtered <- cpm(myDGEList.filtered, log=TRUE)
log2.cpm.filtered.df <- as_tibble(log2.cpm.filtered) 
colnames(log2.cpm.filtered.df) <- sampleLabels
log2.cpm.filtered.df.melt <- melt(log2.cpm.filtered.df)

ggplot(log2.cpm.filtered.df.melt, aes(x=variable, y=value, fill=variable)) +
  geom_violin(trim = FALSE, show.legend = FALSE) +
  stat_summary(fun.y = "median", geom = "point", shape = 124, size = 6, color = "black", show.legend = FALSE) +
  labs(y="log2 expression", x = "sample",
       title="Log2 Counts per Million (CPM)",
       subtitle="filtered, non-normalized",
       caption=paste0("produced on ", Sys.time())) +
  coord_flip()
```

```
# Normalize the data ----
myDGEList.filtered.norm <- calcNormFactors(myDGEList.filtered, method = "TMM")

# use the 'cpm' function from EdgeR to get counts per million from the normalized data
log2.cpm.filtered.norm <- cpm(myDGEList.filtered.norm, log=TRUE)
log2.cpm.filtered.norm.df <- as_tibble(log2.cpm.filtered.norm)
colnames(log2.cpm.filtered.norm.df) <- sampleLabels
log2.cpm.filtered.norm.df.melt <- melt(log2.cpm.filtered.norm.df)

ggplot(log2.cpm.filtered.norm.df.melt, aes(x=variable, y=value, fill=variable)) +
  geom_violin(trim = FALSE, show.legend = FALSE) +
  stat_summary(fun.y = "median", geom = "point", shape = 124, size = 6, color = "black", show.legend = FALSE) +
  labs(y="log2 expression", x = "sample",
       title="Log2 Counts per Million (CPM)",
       subtitle="filtered, TMM normalized",
       caption=paste0("produced on ", Sys.time())) +
  coord_flip()
```

```
# Need to convert the datamatrix to a dataframe, while preserving the rownames as a new column in the dataframe
mydata.df <- as_tibble(log2.cpm.filtered.norm, rownames = "geneSymbol")
colnames(mydata.df) <- c("geneSymbol", sampleLabels)
mydata.melt <- as_tibble(melt(mydata.df))

write_tsv(mydata.df, "normData_CryptoDB_AP2_F_Knockdown.txt") 
#This data was used for GSEA analysis using the Desktop Software GSEA 4.3.2
#Number of permutations is 1000
#Enrichment statistic weighted
#Metric for ranking genes Diff_of_Classes
#All other parameters kept as default
```

Filtering was carried out to remove lowly expressed genes. For filtering, only genes with >= 10 counts per million (CPM) in at least 3 or more samples were kept. This reduced the number of genes from 4023 to 2327.

---

## 6.2 PCA of data after filtering and normalization

```
# Principal component analysis (PCA) -------------
pca.res <- prcomp(t(log2.cpm.filtered.norm), scale.=F, retx=T)
x <- pca.res$rotation 
pc.var<-pca.res$sdev^2 
pc.per<-round(pc.var/sum(pc.var)*100, 1)
pc.per
```

```
##  [1] 19.0 11.9 10.4  9.6  9.1  8.8  7.7  7.1  7.0  5.9  3.4  0.0
```

```
# Visualize the PCA result ------------------
pca.res.df <- as_tibble(pca.res$x)
ggplot(pca.res.df, aes(x=PC1, y=PC2, color=targets$strain_condition)) +
  geom_point(size=8) +
  theme_bw() +
  xlab(paste0("PC1 (",pc.per[1],"%",")")) + 
  ylab(paste0("PC2 (",pc.per[2],"%",")")) +
  labs(title="PCA plot of strains and treatment groups") +
  theme(legend.title = element_blank()) +
  theme(axis.text=element_text(size = 14),
        axis.title = element_text(size=16),
        plot.title=element_text(face = "bold", size = 20),
        legend.text = element_text(size=16))
```

---

## 6.3 Volcano plots of WT and AP2-F DiCre untreated and rapamycin treated parasites

```
# Set up the design matrix ----
groups1 <- relevel(groups1, "AP2_F_rap")
design <- model.matrix(~0 + groups1)
colnames(design) <- levels(groups1)


# Model mean-variance trend and fit linear model to data ----
# Use VOOM function from Limma package to model the mean-variance relationship
v.DGEList.filtered.norm <- voom(myDGEList.filtered.norm, design, plot = TRUE)
```

```
# fit a linear model to your data
fit <- lmFit(v.DGEList.filtered.norm, design)


# Contrast matrix ----
# how do parasites respond to rapamycin treatment? What is the effect of the knockdown?
contrast.matrix <- makeContrasts(AP2F_rap_AP2F_ctrl = AP2_F_rap - AP2_F_control,
                                 WT_rap_WT_ctrl = WT_rap - WT_control,
                                 AP2F_ctrl_WT_ctrl = AP2_F_control - WT_control,
                                 AP2F_rap_WT_rap = AP2_F_rap - WT_rap,
                                 levels=design)


# extract the linear model fit -----
fits <- contrasts.fit(fit, contrast.matrix)
ebFit <- eBayes(fits)

# TopTable to view differentially expressed genes -----

#myTopHits is AP2_F rapamycin versus AP2_F control
myTopHits <- topTable(ebFit, adjust ="BH", coef=1, number=10000, sort.by="logFC")

# convert to a tibble
myTopHits <- as_tibble(myTopHits, rownames = "geneSymbol")


#myTopHits2 is WT rapamycin versus WT control
myTopHits2 <- topTable(ebFit, adjust ="BH", coef=2, number=10000, sort.by="logFC")

# convert to a tibble
myTopHits2 <- as_tibble(myTopHits2, rownames = "geneSymbol")


#myTopHits3 is AP2_F ctrl versus WT control
myTopHits3 <- topTable(ebFit, adjust ="BH", coef=3, number=10000, sort.by="logFC")

# convert to a tibble
myTopHits3 <- as_tibble(myTopHits3, rownames = "geneSymbol")


#myTopHits4 is AP2_F rapamycin versus WT rapamycin
myTopHits4 <- topTable(ebFit, adjust ="BH", coef=4, number=10000, sort.by="logFC")

# convert to a tibble
myTopHits4 <- as_tibble(myTopHits4, rownames = "geneSymbol")


#Pull out genes from final LOPIT paper submission, column tagm.class.pam.pred.lab
crystalloid_body_LOPIT_submission_AP2_F <- subset(myTopHits,
                                                  geneSymbol=="cgd1_1510-RA" |
                                                    geneSymbol=="cgd2_2100-RA" |
                                                    geneSymbol=="cgd2_2110-RA" |
                                                    geneSymbol=="cgd2_790-RA" |
                                                    geneSymbol=="cgd6_313-RA" |
                                                    geneSymbol=="cgd6_820-RA" |
                                                    geneSymbol=="cgd7_1730-RA" |
                                                    geneSymbol=="cgd7_300-RA" |
                                                    geneSymbol=="cgd7_4810-RA" |
                                                    geneSymbol=="cgd7_5140-RA" |
                                                    geneSymbol=="cgd8_4290-RA" |
                                                    geneSymbol=="cgd8_4300-RA" |
                                                    geneSymbol=="cgd8_4310-RA" )


oocyst_wall_LOPIT_submission_AP2_F <- subset(myTopHits,
                                             geneSymbol== "cgd1_3550-RA" |
                                               geneSymbol== "cgd1_800-RA" |
                                               geneSymbol== "cgd2_1590-RA" |
                                               geneSymbol== "cgd2_2510-RA" |
                                               geneSymbol== "cgd2_3040-RA" |
                                               geneSymbol== "cgd2_4350-RA" |
                                               geneSymbol== "cgd2_850-RA" |
                                               geneSymbol== "cgd3_1540-RA" |
                                               geneSymbol== "cgd3_1860-RA" |
                                               geneSymbol== "cgd3_190-RA" |
                                               geneSymbol== "cgd3_3700-RA" |
                                               geneSymbol== "cgd4_3090-RA" |
                                               geneSymbol== "cgd4_500-RA" |
                                               geneSymbol== "cgd4_670-RA" |
                                               geneSymbol== "cgd5_3073-RA" |
                                               geneSymbol== "cgd6_1450-RA" |
                                               geneSymbol== "cgd6_200-RA" |
                                               geneSymbol== "cgd6_2090-RA" |
                                               geneSymbol== "cgd6_210-RA" |
                                               geneSymbol== "cgd6_2470-RA" |
                                               geneSymbol== "cgd6_2900-RA" |
                                               geneSymbol== "cgd6_2920-RA" |
                                               geneSymbol== "cgd6_3730-RA" |
                                               geneSymbol== "cgd6_4440-RA" |
                                               geneSymbol== "cgd6_4640-RA" |
                                               geneSymbol== "cgd6_4840-RA" |
                                               geneSymbol== "cgd6_670-RA" |
                                               geneSymbol== "cgd6_710-RA" |
                                               geneSymbol== "cgd7_180-RA" |
                                               geneSymbol== "cgd7_1800-RA" |
                                               geneSymbol== "cgd7_4310-RA" |
                                               geneSymbol== "cgd7_4560-RA" |
                                               geneSymbol== "cgd7_5150-RA" |
                                               geneSymbol== "cgd7_5400-RA" |
                                               geneSymbol== "cgd7_850-RA" |
                                               geneSymbol== "cgd8_2670-RA" |
                                               geneSymbol== "cgd8_3350-RA" |
                                               geneSymbol== "cgd8_3870-RA" |
                                               geneSymbol== "cgd8_4230-RA" |
                                               geneSymbol== "cgd8_4660-RA" |
                                               geneSymbol== "cgd8_4830-RA" |
                                               geneSymbol== "cgd8_5080-RA" |
                                               geneSymbol== "cgd8_5090-RA" |
                                               geneSymbol== "cgd8_620-RA")

crystalloid_body_LOPIT_submission_WT <- subset(myTopHits2,
                                               geneSymbol=="cgd1_1510-RA" |
                                                 geneSymbol=="cgd2_2100-RA" |
                                                 geneSymbol=="cgd2_2110-RA" |
                                                 geneSymbol=="cgd2_790-RA" |
                                                 geneSymbol=="cgd6_313-RA" |
                                                 geneSymbol=="cgd6_820-RA" |
                                                 geneSymbol=="cgd7_1730-RA" |
                                                 geneSymbol=="cgd7_300-RA" |
                                                 geneSymbol=="cgd7_4810-RA" |
                                                 geneSymbol=="cgd7_5140-RA" |
                                                 geneSymbol=="cgd8_4290-RA" |
                                                 geneSymbol=="cgd8_4300-RA" |
                                                 geneSymbol=="cgd8_4310-RA" )


oocyst_wall_LOPIT_submission_WT <- subset(myTopHits2,
                                          geneSymbol== "cgd1_3550-RA" |
                                            geneSymbol== "cgd1_800-RA" |
                                            geneSymbol== "cgd2_1590-RA" |
                                            geneSymbol== "cgd2_2510-RA" |
                                            geneSymbol== "cgd2_3040-RA" |
                                            geneSymbol== "cgd2_4350-RA" |
                                            geneSymbol== "cgd2_850-RA" |
                                            geneSymbol== "cgd3_1540-RA" |
                                            geneSymbol== "cgd3_1860-RA" |
                                            geneSymbol== "cgd3_190-RA" |
                                            geneSymbol== "cgd3_3700-RA" |
                                            geneSymbol== "cgd4_3090-RA" |
                                            geneSymbol== "cgd4_500-RA" |
                                            geneSymbol== "cgd4_670-RA" |
                                            geneSymbol== "cgd5_3073-RA" |
                                            geneSymbol== "cgd6_1450-RA" |
                                            geneSymbol== "cgd6_200-RA" |
                                            geneSymbol== "cgd6_2090-RA" |
                                            geneSymbol== "cgd6_210-RA" |
                                            geneSymbol== "cgd6_2470-RA" |
                                            geneSymbol== "cgd6_2900-RA" |
                                            geneSymbol== "cgd6_2920-RA" |
                                            geneSymbol== "cgd6_3730-RA" |
                                            geneSymbol== "cgd6_4440-RA" |
                                            geneSymbol== "cgd6_4640-RA" |
                                            geneSymbol== "cgd6_4840-RA" |
                                            geneSymbol== "cgd6_670-RA" |
                                            geneSymbol== "cgd6_710-RA" |
                                            geneSymbol== "cgd7_180-RA" |
                                            geneSymbol== "cgd7_1800-RA" |
                                            geneSymbol== "cgd7_4310-RA" |
                                            geneSymbol== "cgd7_4560-RA" |
                                            geneSymbol== "cgd7_5150-RA" |
                                            geneSymbol== "cgd7_5400-RA" |
                                            geneSymbol== "cgd7_850-RA" |
                                            geneSymbol== "cgd8_2670-RA" |
                                            geneSymbol== "cgd8_3350-RA" |
                                            geneSymbol== "cgd8_3870-RA" |
                                            geneSymbol== "cgd8_4230-RA" |
                                            geneSymbol== "cgd8_4660-RA" |
                                            geneSymbol== "cgd8_4830-RA" |
                                            geneSymbol== "cgd8_5080-RA" |
                                            geneSymbol== "cgd8_5090-RA" |
                                            geneSymbol== "cgd8_620-RA")


#Make a volcano plot for AP2-F DiCre untreated and treated
ggplot(myTopHits, aes(y=-log10(adj.P.Val), x=logFC, text = paste("Symbol:", geneSymbol))) + geom_point(size=4) +
  geom_point(mapping = NULL, oocyst_wall_LOPIT_submission_AP2_F, size = 4, colour = "mediumpurple2", inherit.aes = TRUE) +
  geom_point(mapping = NULL, crystalloid_body_LOPIT_submission_AP2_F, size = 4, colour = "magenta", inherit.aes = TRUE) +
  ylim(-0.5,10) +
  xlim(-4,4) +
  geom_hline(yintercept = -log10(0.01), linetype="dashed", colour="grey", size=0.5) +
  geom_hline(yintercept = -log10(0.05), linetype="dashed", colour="grey", size=1) +
  geom_vline(xintercept = 1, linetype="longdash", colour="grey", size=1) +
  geom_vline(xintercept = -1, linetype="longdash", colour="grey", size=1) +
  theme_bw() +
  theme(axis.text=element_text(size = 14),
        axis.title = element_text(size=16),
        plot.title=element_text(face = "bold", size = 20),
        plot.subtitle = element_text(size=16)) +
  labs(title="Effects of DiCre-mediated knockout of AP2-F")
```

```
#Make a volcano plot for WT untreated and treated
ggplot(myTopHits2, aes(y=-log10(adj.P.Val), x=logFC, text = paste("Symbol:", geneSymbol))) +
  geom_point(size=4) +
  geom_point(mapping = NULL, oocyst_wall_LOPIT_submission_WT, size = 4, colour = "mediumpurple2", inherit.aes = TRUE) +
  geom_point(mapping = NULL, crystalloid_body_LOPIT_submission_WT, size = 4, colour = "magenta", inherit.aes = TRUE) +
  ylim(-0.5,10) +
  xlim(-4,4) +
  geom_hline(yintercept = -log10(0.01), linetype="dashed", colour="grey", size=0.5) +
  geom_hline(yintercept = -log10(0.05), linetype="dashed", colour="grey", size=1) +
  geom_vline(xintercept = 1, linetype="longdash", colour="grey", size=1) +
  geom_vline(xintercept = -1, linetype="longdash", colour="grey", size=1) +
  theme_bw() +
  theme(axis.text=element_text(size = 14),
        axis.title = element_text(size=16),
        plot.title=element_text(face = "bold", size = 20)) +
  labs(title="Effects of rapamycin treatment on WT")
```

---

## 6.4 Table of differentially expressed genes

To identify differentially expressed genes, precision weights were first applied to each gene based on its mean-variance relationship using VOOM, then data was normalized using the TMM method in EdgeR. Linear modeling and bayesian stats were employed via Limma to find genes that were up- or down-regulated by a log2-transformed fold change of 1 or more, with a false-discovery rate (FDR) of 0.01.

```
# decideTests to pull out the DEGs ----
results <- decideTests(ebFit, method="global", adjust.method="BH", p.value=0.01, lfc=1)

# retrieve expression data for DEGs ----
head(v.DGEList.filtered.norm$E)
colnames(v.DGEList.filtered.norm$E) <- sampleLabels

#This gives all the DiffGenes, regardless of significance between samples (only needs to be significant in one)
diffGenes <- v.DGEList.filtered.norm$E[results[,1] !=0 | results[,2] !=0 | results[,3] !=0 | results[,4] !=0,]
head(diffGenes)
dim(diffGenes)
#convert DEGs to a dataframe using as_tibble
diffGenes.df <- as_tibble(diffGenes, rownames = "geneSymbol")


#Adding averages to Diff Genes
#This is where to make new files for gene expression log-fold changes
Differential_genes_avg.df <- mutate(diffGenes.df,
                                    WT_Ctrl.AVG = (WT_Ctrl_rep_1 + WT_Ctrl_rep_2 + WT_Ctrl_rep_3)/3,
                                    WT_Rap.AVG = (WT_Rap_rep_1 + WT_Rap_rep_2 + WT_Rap_rep_3)/3,
                                    AP2_F_Ctrl.AVG = (AP2_F_Ctrl_rep_1 + AP2_F_Ctrl_rep_2 + AP2_F_Ctrl_rep_3)/3,
                                    AP2_F_Rap.AVG = (AP2_F_Rap_rep_1 + AP2_F_Rap_rep_2 + AP2_F_Rap_rep_3)/3,
                                    
                                    
                                    #now make columns comparing each of the averages above 
                                    #do as rapamycin likely downregulates, so put control first
                                    
                                    LogFC.AP2F_rap_vs_AP2F_ctrl = (AP2_F_Rap.AVG - AP2_F_Ctrl.AVG),
                                    LogFC.WT_rap_vs_WT_ctrl = (WT_Rap.AVG - WT_Ctrl.AVG),
                                    LogFC.AP2F_ctrl_vs_WT_ctrl = (AP2_F_Ctrl.AVG - WT_Ctrl.AVG),
                                    LogFC.AP2F_rap_vs_WT_rap = (AP2_F_Rap.AVG - WT_Rap.AVG)) %>%
  mutate_if(is.numeric, round, 3)

#This is Supplemental File 1
write_tsv(Differential_genes_avg.df, "2021_5_20_Differential_genes_avg_CryptoDB_50_AP2F_knockdown_DiCre.txt")


#write DEGs to a file
write_tsv(diffGenes.df,"2021_5_20_DiffGenes_CryptoDB_50_LFC1.txt") 

#Check different ones, add as new spreadsheets to Supplemental File 1
#AP2F_rap_vs_AP2F_ctrl
diffGenes_AP2F <- v.DGEList.filtered.norm$E[results[,1] !=0,]
head(diffGenes_AP2F)
dim(diffGenes_AP2F)
#convert DEGs to a dataframe using as_tibble
diffGenes_AP2F.df <- as_tibble(diffGenes_AP2F, rownames = "geneSymbol")


Differential_genes_avg_AP2F.df <- mutate(diffGenes_AP2F.df,
                                         AP2_F_Ctrl.AVG = (AP2_F_Ctrl_rep_1 + AP2_F_Ctrl_rep_2 + AP2_F_Ctrl_rep_3)/3,
                                         AP2_F_Rap.AVG = (AP2_F_Rap_rep_1 + AP2_F_Rap_rep_2 + AP2_F_Rap_rep_3)/3,
                                         #now make columns comparing each of the averages above 
                                         LogFC.AP2F_rap_vs_AP2F_ctrl = (AP2_F_Rap.AVG - AP2_F_Ctrl.AVG)) %>%
  mutate_if(is.numeric, round, 3)


write_tsv(Differential_genes_avg_AP2F.df, "2021_5_20_Differential_genes_avg_AP2F_only_LFC1.txt")


#WT_rap_vs_WT_ctrl
diffGenes_WT <- v.DGEList.filtered.norm$E[results[,2] !=0,]
head(diffGenes_WT)
dim(diffGenes_WT)
#convert DEGs to a dataframe using as_tibble
diffGenes_WT.df <- as_tibble(diffGenes_WT, rownames = "geneSymbol")

#Error, only giving cgd2_4100 but no gene ID, no dims (not significant - adjusted p value just above 0.01)
Differential_genes_avg_WT.df <- mutate(diffGenes_WT.df,
                                       WT_Ctrl.AVG = (WT_Ctrl_rep_1 + WT_Ctrl_rep_2 + WT_Ctrl_rep_3)/3,
                                       WT_Rap.AVG = (WT_Rap_rep_1 + WT_Rap_rep_2 + WT_Rap_rep_3)/3,
                                       #now make columns comparing each of the averages above 
                                       LogFC.WT_rap_vs_WT_ctrl = (WT_Rap.AVG - WT_Ctrl.AVG)) %>%
  mutate_if(is.numeric, round, 3)


write_tsv(Differential_genes_avg_WT.df, "2021_5_20_Differential_genes_avg_WT_only_LFC1.txt")


#AP2F_ctrl_vs_WT_ctrl
diffGenes_ctrl <- v.DGEList.filtered.norm$E[results[,3] !=0,]
head(diffGenes_ctrl)
dim(diffGenes_ctrl)
#convert DEGs to a dataframe using as_tibble
diffGenes_ctrl.df <- as_tibble(diffGenes_ctrl, rownames = "geneSymbol")

Differential_genes_avg_ctrl.df <- mutate(diffGenes_ctrl.df,
                                         WT_Ctrl.AVG = (WT_Ctrl_rep_1 + WT_Ctrl_rep_2 + WT_Ctrl_rep_3)/3,
                                         AP2_F_Ctrl.AVG = (AP2_F_Ctrl_rep_1 + AP2_F_Ctrl_rep_2 + AP2_F_Ctrl_rep_3)/3,
                                         #now make columns comparing each of the averages above 
                                         LogFC.AP2F_ctrl_vs_WT_ctrl = (AP2_F_Ctrl.AVG - WT_Ctrl.AVG)) %>%
  mutate_if(is.numeric, round, 3)


write_tsv(Differential_genes_avg_ctrl.df, "2021_5_25_Differential_genes_avg_ctrl_only_LFC1.txt")


#AP2F_rap_vs_WT_rap
diffGenes_rap <- v.DGEList.filtered.norm$E[results[,4] !=0,]
head(diffGenes_rap)
dim(diffGenes_rap)
#convert DEGs to a dataframe using as_tibble
diffGenes_rap.df <- as_tibble(diffGenes_rap, rownames = "geneSymbol")

Differential_genes_avg_rap.df <- mutate(diffGenes_rap.df,
                                        WT_Rap.AVG = (WT_Rap_rep_1 + WT_Rap_rep_2 + WT_Rap_rep_3)/3,
                                        AP2_F_Rap.AVG = (AP2_F_Rap_rep_1 + AP2_F_Rap_rep_2 + AP2_F_Rap_rep_3)/3,
                                        #now make columns comparing each of the averages above 
                                        LogFC.AP2F_rap_vs_WT_rap = (AP2_F_Rap.AVG - WT_Rap.AVG)) %>%
  mutate_if(is.numeric, round, 3)


write_tsv(Differential_genes_avg_rap.df, "2021_5_25_Differential_genes_avg_rap_only_LFC1.txt")
```

---

# 7 Session info

The output from running ‘sessionInfo’ is shown below and details all packages and versions necessary to reproduce the results in this report.

```
sessionInfo()
```

```
## R version 4.0.3 (2020-10-10)
## Platform: x86_64-apple-darwin17.0 (64-bit)
## Running under: macOS Catalina 10.15.7
## 
## Matrix products: default
## BLAS:   /Library/Frameworks/R.framework/Versions/4.0/Resources/lib/libRblas.dylib
## LAPACK: /Library/Frameworks/R.framework/Versions/4.0/Resources/lib/libRlapack.dylib
## 
## locale:
## [1] en_US.UTF-8/en_US.UTF-8/en_US.UTF-8/C/en_US.UTF-8/en_US.UTF-8
## 
## attached base packages:
## [1] stats     graphics  grDevices utils     datasets  methods   base     
## 
## other attached packages:
##  [1] skimr_2.1.4        plotly_4.10.1      gt_0.8.0           DT_0.26           
##  [5] matrixStats_0.61.0 edgeR_3.32.1       limma_3.46.0       genefilter_1.72.1 
##  [9] reshape2_1.4.4     RColorBrewer_1.1-3 hrbrthemes_0.8.0   biomaRt_2.46.3    
## [13] tximport_1.18.0    forcats_0.5.2      stringr_1.4.1      dplyr_1.0.8       
## [17] purrr_0.3.4        readr_2.1.2        tidyr_1.2.0        tibble_3.1.8      
## [21] ggplot2_3.3.6      tidyverse_1.3.2    knitr_1.34         tinytex_0.42      
## [25] rmarkdown_2.18    
## 
## loaded via a namespace (and not attached):
##   [1] googledrive_2.0.0    colorspace_2.1-0     ellipsis_0.3.2      
##   [4] base64enc_0.1-4      fs_1.5.2             rstudioapi_0.14     
##   [7] farver_2.1.1         bit64_4.0.5          AnnotationDbi_1.52.0
##  [10] fansi_1.0.3          lubridate_1.8.0      xml2_1.3.3          
##  [13] splines_4.0.3        extrafont_0.18       cachem_1.0.6        
##  [16] jsonlite_1.8.0       broom_1.0.1          Rttf2pt1_1.3.10     
##  [19] annotate_1.68.0      dbplyr_2.1.1         compiler_4.0.3      
##  [22] httr_1.4.4           backports_1.4.1      assertthat_0.2.1    
##  [25] Matrix_1.4-1         fastmap_1.1.0        lazyeval_0.2.2      
##  [28] gargle_1.2.1         cli_3.2.0            htmltools_0.5.3     
##  [31] prettyunits_1.1.1    tools_4.0.3          gtable_0.3.1        
##  [34] glue_1.6.2           rappdirs_0.3.3       Rcpp_1.0.9          
##  [37] Biobase_2.50.0       cellranger_1.1.0     jquerylib_0.1.4     
##  [40] rhdf5filters_1.2.1   vctrs_0.4.0          extrafontdb_1.0     
##  [43] xfun_0.30            rvest_1.0.3          lifecycle_1.0.1     
##  [46] XML_3.99-0.9         googlesheets4_1.0.1  scales_1.2.1        
##  [49] vroom_1.5.7          hms_1.1.2            parallel_4.0.3      
##  [52] rhdf5_2.34.0         yaml_2.3.5           curl_4.3.2          
##  [55] memoise_2.0.1        gdtools_0.2.4        sass_0.4.1          
##  [58] stringi_1.7.8        RSQLite_2.2.12       highr_0.9           
##  [61] S4Vectors_0.28.1     BiocGenerics_0.36.1  repr_1.1.4          
##  [64] rlang_1.0.2          pkgconfig_2.0.3      systemfonts_1.0.4   
##  [67] evaluate_0.18        lattice_0.20-45      Rhdf5lib_1.12.1     
##  [70] labeling_0.4.2       htmlwidgets_1.5.4    bit_4.0.4           
##  [73] tidyselect_1.1.2     plyr_1.8.7           magrittr_2.0.3      
##  [76] R6_2.5.1             IRanges_2.24.1       generics_0.1.3      
##  [79] DBI_1.1.3            pillar_1.8.1         haven_2.4.3         
##  [82] withr_2.5.0          survival_3.3-1       modelr_0.1.8        
##  [85] crayon_1.5.2         utf8_1.2.2           BiocFileCache_1.14.0
##  [88] tzdb_0.3.0           progress_1.2.2       locfit_1.5-9.4      
##  [91] grid_4.0.3           readxl_1.4.0         data.table_1.14.2   
##  [94] blob_1.2.3           reprex_2.0.2         digest_0.6.29       
##  [97] xtable_1.8-6         openssl_2.0.0        stats4_4.0.3        
## [100] munsell_0.5.0        viridisLite_0.4.1    bslib_0.4.1         
## [103] askpass_1.1
```
